# Identifying influential neighbors in animal flocking

**DOI:** 10.1371/journal.pcbi.1005822

**Published:** 2017-11-21

**Authors:** Li Jiang, Luca Giuggioli, Andrea Perna, Ramón Escobedo, Valentin Lecheval, Clément Sire, Zhangang Han, Guy Theraulaz

**Affiliations:** 1 School of Systems Science, Beijing Normal University, Beijing, China; 2 Centre de Recherches sur la Cognition Animale, Centre de Biologie Intégrative (CBI), Centre National de la Recherche Scientifique (CNRS), Université Paul Sabatier (UPS), Toulouse, France; 3 Bristol Centre for Complexity Sciences, Department of Engineering Mathematics and School of Biological Sciences, University of Bristol, Bristol, United Kingdom; 4 Life Sciences, Roehampton University, London, United Kingdom; 5 Groningen Institute for Evolutionary Life Sciences, University of Groningen, Centre for Life Sciences, Groningen, The Netherlands; 6 Laboratoire de Physique Théorique, CNRS & Université de Toulouse (UPS), Toulouse, France; Oxford, UNITED KINGDOM

## Abstract

Schools of fish and flocks of birds can move together in synchrony and decide on new directions of movement in a seamless way. This is possible because group members constantly share directional information with their neighbors. Although detecting the directionality of other group members is known to be important to maintain cohesion, it is not clear how many neighbors each individual can simultaneously track and pay attention to, and what the spatial distribution of these influential neighbors is. Here, we address these questions on shoals of *Hemigrammus rhodostomus*, a species of fish exhibiting strong schooling behavior. We adopt a data-driven analysis technique based on the study of short-term directional correlations to identify which neighbors have the strongest influence over the participation of an individual in a collective U-turn event. We find that fish mainly react to one or two neighbors at a time. Moreover, we find no correlation between the distance rank of a neighbor and its likelihood to be influential. We interpret our results in terms of fish allocating sequential and selective attention to their neighbors.

## Author summary

Schooling fish exhibit impressive group-level coordination in which multiple individuals move together in a seamless way. This is possible because each individual in the group responds to the movement of other group members. But how many individuals does each fish pay attention to? Which are the influential neighbors? It is necessary to answer these questions in order to understand how directional information propagates across a group. Our research shows that in the rummy-nose tetra species there is a limited number of influential neighbors which are not necessarily the closest ones.

## Introduction

Collective motion phenomena such as swarming, flocking and schooling behavior have been observed in a large variety of animal species ranging from bacteria to humans [1]. Several theoretical models have been proposed to explain how such large scale coordination patterns emerge from “microscopic level” interaction rules among individual animals [2–7]. These models have been instrumental in improving our understanding of collective motion in real animal groups by providing an indication of which interaction mechanisms are sufficient to reproduce realistic patterns of collective behavior. In particular, most models agree on the fact that two types of interaction are responsible for maintaining group cohesion to achieve coherent collective motion: attraction and alignment.

More recent improvements in remote sensing and video-tracking technologies [8–10] have made possible to automate data collection and test directly theoretical models against highly resolved empirical movement data in various species. Generally, these studies have confirmed the importance that attraction and alignment behavior play in the formation and maintenance of collective movement patterns [11–15]. However, there is a less clear scientific consensus about how these interaction rules are implemented in the sensory-motor responses of individuals. This lack of agreement underscores the importance of answering the following question: how do individuals mediate interactions with multiple neighbors? [16].

Specifically, theoretical studies have postulated a number of factors that are likely to affect the probability and intensity of interactions: distance (metric neighborhood) [2–7], position rank (topological neighborhood) [17], projected size (visual neighborhood) [18–20], and spatial arrangement around a focal individual (Voronoi neighborhood) [13]. Each of these different definitions of influential neighborhood is supported to some extent by computational models and empirical observations.

Rather than siding with one or more of the proposed neighborhood definitions, we adopt a fully data-driven approach with minimalist modeling assumptions. The simplest hypothesis consists of assuming that fish copy the actions of their neighbors, but not instantaneously: the fish reaction takes time to process sensory information and to trigger the appropriate behavioral response. Those assumptions impose a temporal constraint given by the sequential occurrence of the perception of the neighbors’ actions, and the movement response [21, 22]. We thus assume that animals following a particular neighbor in a new direction are subject to a time-delay when copying the heading of influential neighbors.

Considerable work has already appeared on the identification of these time-delays. The delays with which individuals align with each other have in fact been exploited to determine social hierarchies in animal groups, as shown, *e.g.*, for pigeon flocks [23], where the leadership network is constructed with link weights given by the delay for which pairwise angle correlation is maximal. Improvements on how to identify such delays from movement data have proposed the use of time-dependence in pairwise angle correlation [24]. A computational analysis, based on similarities between trajectories (Fréchet distance), has also been proposed and implemented in a visual analytic tool [25]. A different approach has made use of a time-ordering procedure on the pairwise angle correlation to determine temporary leader/follower relations in foraging pairs of echolocating bats [26]. The analysis of the bat trajectories was instrumental in identifying transient leadership and coupling it to sensory biases of the species. However, only pairs of individuals were considered and group influence on individual behavior was not investigated.

Since identifying influential neighbors is key to unravel the mechanisms of interaction, there is a need in collective behavior studies to establish transient leadership from the dynamics of the individual trajectories. One way to bridge this gap consists of determining who are those influential individuals whose heading is being copied more closely by others, how many of such influential neighbors exist, and where are located in the group.

Fish have the ability to choose not only when to copy the heading of another individual, but also the extent to which this heading is copied, that is the similarity and the pace at which fish match the trajectory’s curvature of another individual [11, 27]. The closer two (or more) fish are to this matching, the more aligned they are (even if with some delay), and the more faithfully they are following the movement path of the transient leader.

Here, we introduce a procedure that allows us to identify the influential neighbors of fish moving in a group, and we test it along a series of experiments in groups of two and five individuals of the freshwater tropical fish *Hemigrammus rhodostomus* swimming in a ring-shaped tank (see details in Materials and methods). In this set-up, fish swim in a highly synchronized and polarized manner, and can only head in two directions, clockwise or anticlockwise, regularly switching from one to the other. We base our procedure for identifying influential neighbors on time-dependent directional correlations between fish, focussing our analysis on the interactions that occur during these collective U-turns. Indeed, during U-turns, fish have to make a substantial change of direction to reverse their heading, making easier the extraction of the correlation resulting from the direct interactions between individuals rather than other incidental correlations, *e.g.*, their channeled motion in the ring-shaped tank. Moreover, as correlation does not imply causal influence, we need to control for potential spurious correlations. We do so by constructing a null model of collective U-turns to show that the patterns of interaction observed in the experiments are not due to random processes.

## Results

### Dynamics of collective U-turns

*Hemigrammus rhodostomus* performs burst-and-coast swimming behavior that consists of sudden heading changes combined with brief accelerations followed by quasi-passive, straight decelerations [15]. Moreover, fish spend most of their time swimming in a single group along the wall of the tank. Fish regularly change their position within the group [28], so that every individual fish can be found at the front of the group.

**Fig 1:**
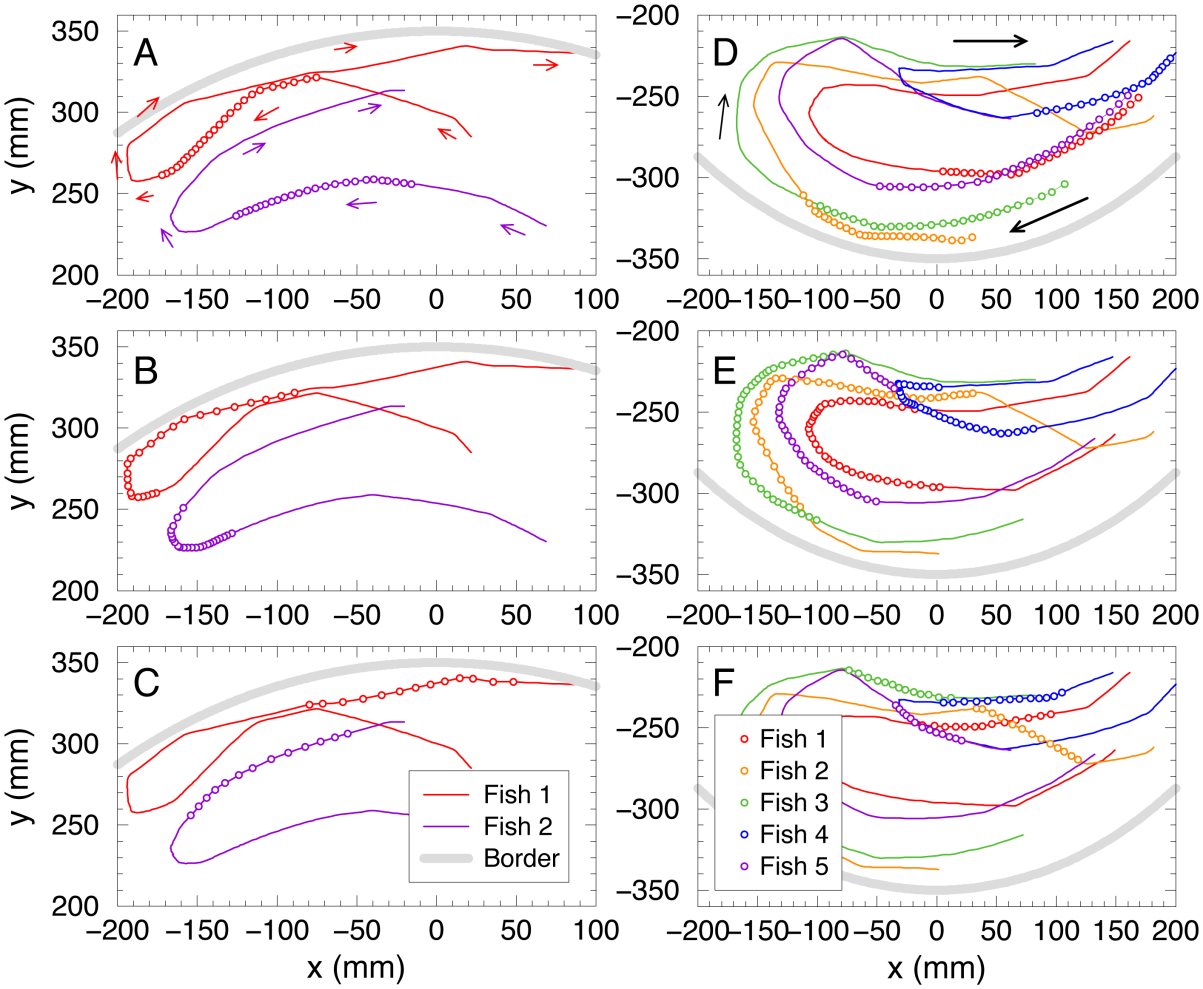
Collective U-turns in groups of two and five fish. Fish trajectories (solid lines) with successive positions (circles) equispaced in time every 0.04s. (ABC): *N* = 2, (DEF): *N* = 5. The top row (AD) displays the collective U-turn one second before it starts, *t* ∈ [*t*_*s*_ − 1 s, *t*_*s*_], where *t*_*s*_ denotes the time at which the collective U-turn starts. The middle row (BE) displays the collective U-turn, *t* ∈ [*t*_*s*_, *t*_*e*_], where *t*_*e*_ denotes the end time of the collective U-turn. The bottom row (CF) displays the movement data 0.5 s after collective U-turn’s end, *t* ∈ [*t*_*e*_, *t*_*e*_ + 0.5 s]. For visual convenience solid lines indicate the actual fish trajectories before *t*_*s*_ − 1 s and after *t*_*e*_ + 0.5 s. Arrows indicate the direction of motion. The grey thick line represents the tank border of radius 35 cm.

A typical collective U-turn event starts with the spontaneous turnaround of a single fish (hereafter called the initiator), mostly located at the front of the group [28]. This sudden change of behavior triggers a collective reaction in which all the other individuals in the group make a U-turn themselves, so that, after a short transient, all individuals adopt the same final direction of motion as the initiator. Overall, we analyzed 1586 U-turns of which 1111 were observed in groups of 2 fish and 475 in groups of 5 fish. Fig 1 shows two examples of collective U-turns in groups of *N* = 2 (left column, panels ABC) and *N* = 5 fish (right column, panels DEF; see also supplementary S8 Fig and supplementary S1 and S2 Videos in the Supplementary Information).

Fig 1A shows a first fish *F*_1_ (red color) swimming close to the upper-left region of the tank, followed by a second fish *F*_2_ (purple color) at a distance *d*_12_ ≈ 8.5 cm, swimming in the same direction. Right before the U-turn starts (Fig 1A), fish *F*_1_ reduces its speed (circles become closer to each other), the distance *d*_12_ decreases (to ≈ 5.1 cm), and *F*_2_ also reduces its speed. Then, both fish perform a change of direction which lasts about 1 second and during which fish *F*_2_ clearly follows fish *F*_1_ (see the corresponding circles at each instant of time in Fig 1B). Once the U-turn is completed (Fig 1C), *F*_1_ accelerates again, and so does *F*_2_, which also adopts the direction of motion of *F*_1_. The distance *d*_12_ increases again (≈ 9.5 cm), due to the larger velocities, and remains of the same order along the depicted trajectory.

The situation is less clear when we try to describe collective U-turns in larger groups. Fig 1D, 1E and 1F show a collective U-turn for the case where *N* = 5. Before the U-turn, fish *F*_2_ (orange) seems to be the fish that the rest of the group follows, the first circle of its trajectory being the most advanced one in the direction of motion. In fact, a position order can be inferred from Fig 1D: *F*_2_, *F*_3_, *F*_5_, *F*_1_ and *F*_4_. However, it is rather complicated to extract from Panel E a precise information about which fish is the initiator of the U-turn, in which order the other fish follow, and therefore, who is influencing whom, especially if time-delays and reaction times are taken into account. The same happens with the information about fish’s positions after the U-turn, provided by Panel F.

**Fig 2:**
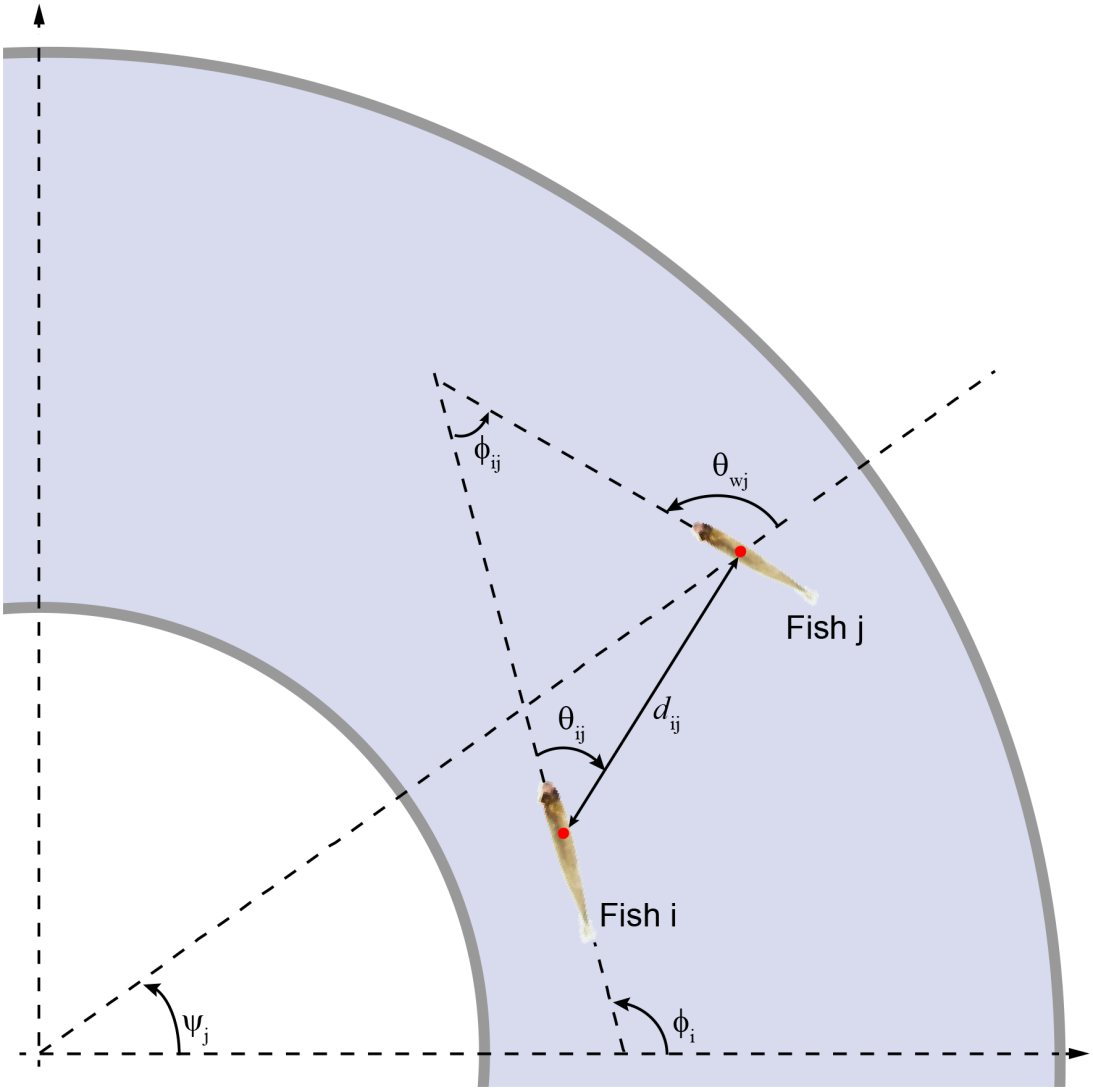
Angles and lengths characterizing the relative position of two fish. Angle *ψ*_*j*_ denotes the angular position of fish *F*_*j*_ with respect to the horizontal (positive values fixed in the anticlockwise direction); angle *ϕ*_*i*_ is the heading of fish *F*_*i*_; *θ*_*wi*_ is the angle of incidence of fish *F*_*i*_ with respect to the outer wall; *d*_*ij*_ is the distance between *F*_*i*_ and *F*_*j*_; *θ*_*ij*_ is the viewing angle of *F*_*i*_ with respect to *F*_*j*_ (not necessarily equal to *θ*_*ji*_), and *ϕ*_*ij*_ = *ϕ*_*j*_ − *ϕ*_*i*_ is the heading difference of *F*_*i*_ with respect to *F*_*j*_.

In order to describe rigorously the individual behavior of the *N* fish during a U-turn, we introduce the angle *ϕ*_*i*_(*t*) as an instantaneous measure of the direction of motion of a fish *F*_*i*_; see Fig 2. We assume that the instantaneous heading of a fish *F*_*i*_ can be defined in terms of the velocity vector ${\vec{v}}_{i}\left(t\right)$, so that ${\vec{v}}_{i}=\left(\cos\phi_{i},\sin\phi_{i}\right)∥{\vec{v}}_{i}∥$. The heading of a fish *ϕ*_*i*_ allows us to characterize the angle of incidence of the fish relative to the wall, *θ*_*wi*_ = *ϕ*_*i*_ − *ψ*_*i*_, where *ψ*_*i*_ is the angle formed by the position vector of the fish with the horizontal line (see Fig 2). The angle of incidence *θ*_*wi*_ is an individual measure that doesn’t depend on the heading of another fish. When a fish *F*_*i*_ is swimming along the wall, the value of *θ*_*wi*_ is around ±90° (we choose, by convention, the positive sign for the anticlockwise angle). In our experiments, most of the time the absolute value of the angle of incidence is close to 90°; equivalently, |sin(*θ*_*wi*_(*t*))| ≈ 1. When the motion is perpendicular to the wall, the incidence is zero if the fish points towards the wall (*θ*_*wi*_ = 0°), and maximal if the fish points towards the center of the tank (*θ*_*wi*_ = 180°); in both cases, sin(*θ*_*wi*_(*t*)) = 0.

The change of sign of angle *θ*_*wi*_ can serve as an indicator that a U-turn has taken place. In fact, this allows us to delimit the individual U-turns with precision and, consequently, to determine the start and the end of a collective U-turn.

We define the *start* and *end* times *t*_*s*,*i*_ and *t*_*e*,*i*_ of the individual U-turn of fish *F*_*i*_ in terms of the absolute value of the angle of incidence, |*θ*_*wi*_(*t*)|. Once a U-turn has been detected, we obtain the time *t*_*s*,*i*_ at which |*θ*_*wi*_(*t*)| has decreased (from approximately 90°) below a given threshold ${\bar{\theta }}_{s}$, and the time *t*_*e*,*i*_ at which |*θ*_*wi*_(*t*)| has increased again and is above another given threshold ${\bar{\theta }}_{e}$ (see Materials and methods for more details).

Thus, the start of a collective U-turn is determined by the time *t*_*s*_ at which the first individual U-turn starts, while the end of a collective U-turn is given by the time *t*_*e*_ at which the last individual U-turn finishes. That is:

$$\begin{array}{c} t_{s}=\min_{i=1,\ldots ,N}\{t_{s,i}\},\;t_{e}=\max_{i=1,\ldots ,N}\{t_{e,i}\}. \end{array}$$ (1)

For each collective U-turn, we have made a convenient time shift so that *t*_*s*_ = 0. Then, *t*_*e*_ denotes not only the end time but also the duration of the collective U-turn.

We also introduce an instantaneous measure of how similar the direction of motion of individual fish are across the group. We define the instantaneous group polarization *P*(*t*) as the following function of normalized fish velocity vectors:

$$\begin{array}{c} P\left(t\right)=\frac{1}{N}∥\sum_{i=1}^{N}{\vec{e}}_{i}\left(t\right)∥, \end{array}$$ (2)

where ${\vec{e}}_{i}={\vec{v}}_{i}/∥{\vec{v}}_{i}∥$. When all the fish have the same direction then the polarization is maximal and *P*(*t*) = 1. The minimum value *P*(*t*) = 0 is reached instead when the velocity vectors cancel.

**Fig 3:**
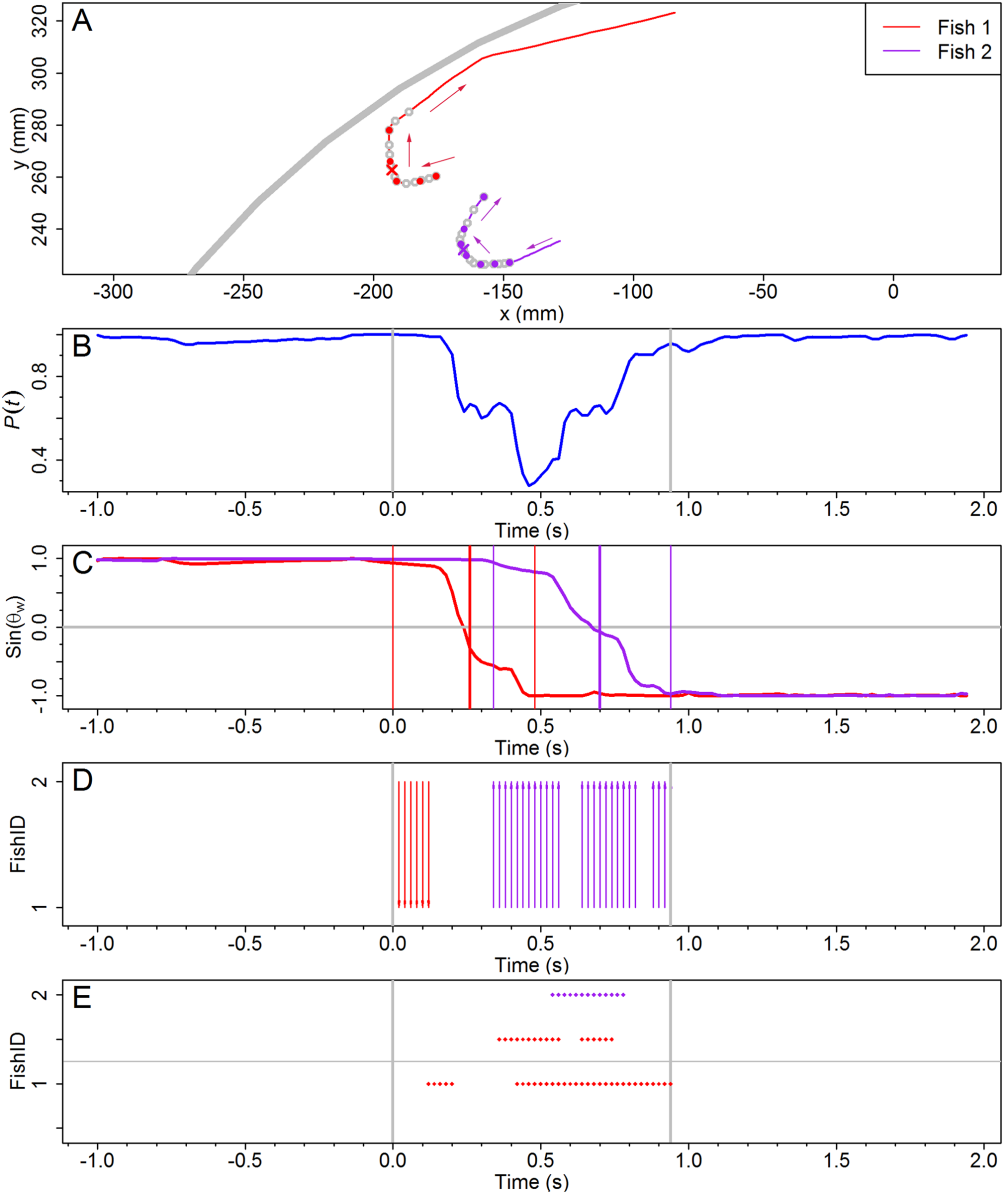
Spatial and temporal dynamics of a collective U-turn for *N* = 2. (A) Individual fish trajectories in the tank during the U-turn. Each individual is represented by a unique color. The temporal sequence is indicated by circles equally spaced over time with a time-step of 0.04 s (empty circles) and 0.1 s (filled circles). Arrows denote direction of motion. Grey wide line is the tank’s border. (B) Group polarization *P*(*t*), with a minimum value *P*_min_ ≈ 0.27 reached at *t* ≈ 0.46 s. (C) Sine of the angle of incidence of fish to the wall: when parallel to the wall, sin(*θ*_*w*_) = 1 (anti-clockwise direction) or sin(*θ*_*w*_) = −1 (clockwise). The three vertical lines of each color indicate for each fish the beginning, the middle and the end of its U-turn, with the middle representing the time when a fish has finally reversed its original direction. (D) Interaction with influential neighbors: arrows point from influential neighbors to the focal fish and with the same color as the focal fish. (E) Fish bursting activity and their influential neighbors. Dots at *i* = 1, 2 correspond to bursting activity, blank corresponds to coasting. Dots at *i* − 0.5 represent bursting activity of the neighbor influencing fish *i*.

**Fig 4:**
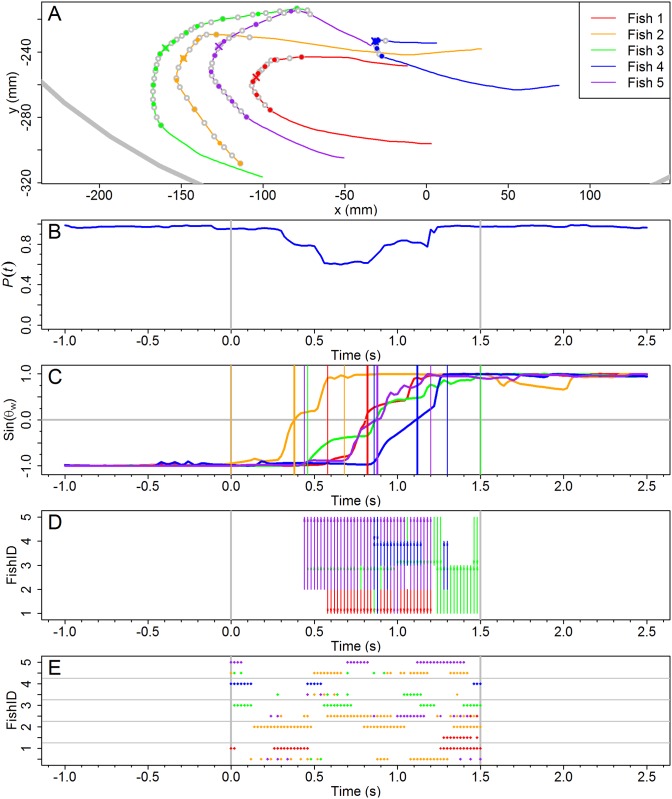
Spatial and temporal dynamics of a collective U-turn for *N* = 5. The displayed temporal sequence is drawn from the fish trajectories one second before the U-turn begins till one second after its end. Symbols in all panels are the same as in Fig 3. (A) Individuals trajectories in the tank during the U-turn. (B) Group polarization with a minimum value *P*_min_ ≈ 0.59 reached at *t* ≈ 0.66 s. (C) Sine of the angle of incidence of fish to the wall *θ*_*w*_. The three vertical lines of each color indicate for each fish the beginning, the middle and the end of its U-turn. Here the middle time means the instant where sin(*θ*_*w*_) = 0. (D) Interactions with influential neighbors: arrows point from influential neighbors to the focal fish and with the same color as the focal fish. (E) Fish bursting activity and their influential neighbors. If there is more than one influential neighbor, *F*_*j*_ with largest index value *j* is shown. Grey lines in Panels BCDE denote the start and end of the collective U-turn.

Figs 3 and 4 depict the two U-turns introduced in Fig 1, in terms of the polarization *P*(*t*) and the sine of the angle of incidence of each fish with respect to the outer wall *θ*_*wi*_(*t*). The duration of the two illustrated collective U-turns is *t*_*e*_ = 0.94 s for *N* = 2 and *t*_*e*_ = 1.5 s for *N* = 5.

For both group sizes, the group polarization (Figs 3B and 4B) before and after the U-turn is quite close to 1, showing that before and after the collective U-turn, all individual fish maintain essentially the same common direction. During the U-turn, the polarization decreases, describing a sharp V-form with a minimum at *P*(*t*) ≈ 0.27 for *N* = 2 and *P*(*t*) ≈ 0.60 for *N* = 5. The minimum is reached at approximately half the duration of the collective U-turn, *t*_*m*_ = (*t*_*s*_ + *t*_*e*_)/2: *t*_*m*_ = 0.47 s for *N* = 2 and *t*_*m*_ = 0.75 s for *N* = 5.

Figs 3C and 4C show the change of direction individually for each fish in both U-turns: from anticlockwise to clockwise direction for *N* = 2, and vice versa for *N* = 5. Fig 3C clearly indicates that at *t* ≈ 0.3 s, the fish *F*_1_ has almost completed its individual U-turn, while *F*_2_ has just started to change direction: sin(*θ*_*w*2_(0.3)) ≈ 0.98, while sin(*θ*_*w*1_(0.3)) ≈ −0.5.

In Fig 4C, a similar ordering can be inferred from the times of departure from the bottom line at ordinate sin(*θ*_*wi*_) = −1 + *δ*, where *δ* > 0 is a small parameter with respect to the range of ordinate values; we used *δ* = 0.1. Thus, the order is 2-3-1-5-4. However, the order in which individual fish change the sign of their angle of incidence *θ*_*wi*_ is different, 2-1-3-5-4, and also different is the arrival order to the top line at ordinate sin(*θ*_*wi*_) = 1 − *δ*: 2-5-1-4-3. Moreover, some of these departure and arrival times are almost identical (see, *e.g.*, *F*_1_ and *F*_4_), and the behavior of the fish during the U-turn is completely different. These difficulties in establishing a consistent order show that another criterion is necessary to identify the relation of influence between fish.

We have based our criterion to decide if a fish is an influential neighbor of another fish on the average value of the time-dependent directional correlation between the two fish along a time window.

For each pair of fish *F*_*i*_ and *F*_*j*_, we define the directional correlation *H*_*ij*_ as a function of the heading of *F*_*i*_ evaluated at time *t* and the heading of *F*_*j*_ evaluated at a delayed time *t* − *τ*, where *τ* is the time-delay [26]:

$$\begin{array}{c} H_{ij}\left(t,\tau \right)={\vec{e}}_{i}\left(t\right)\cdot {\vec{e}}_{j}\left(t-\tau \right). \end{array}$$ (3)

The function *H*_*ij*_(*t*, *τ*) is in fact the cosine of the angle formed by the headings ${\vec{e}}_{i}\left(t\right)$ and ${\vec{e}}_{j}\left(t-\tau \right)$, and is a measure of how aligned is fish *F*_*i*_ at time *t* with fish *F*_*j*_ at time *t* − *τ*. The values of *H*_*ij*_(*t*, *τ*) are between −1 (when fish swim in opposite directions) and 1 (when fish have the same direction), and equals zero when fish have perpendicular directions.

By averaging *H*_*ij*_(*t*, *τ*) along a time-window of length (2*w* + 1)Δ*t*, we are able to quantify how much the focal fish *F*_*i*_ is copying the moving direction of its neighbor with a time-delay *τ* by means of the following function [26]

$$\begin{array}{c} C_{ij}\left(t,\tau ,w\right)=\frac{1}{2w+1}\sum_{k=-w}^{w}H_{ij}\left(t+t_{k},\tau \right), \end{array}$$ (4)

where *t*_*k*_ = *k*Δ*t* (the time-step in our experiments is Δ*t* = 0.02s). The time-window parameter length *w* has been determined by means of a sensitivity analysis (pairwise similarity matrix), finding that *w* = 2 yields the more satisfactory results; see Section “Parameter selection” in Materials and methods and S5 Fig.

The average directional correlation *C*_*ij*_(*t*, *τ*, *w*) allows us to characterize a fish *F*_*j*_ as an influential neighbor of a focal fish *F*_*i*_ at time *t* with time-delay *τ*, if the value of *C*_*ij*_(*t*, *τ*, *w*) is larger than a given threshold *C*_min_. Details on how *w* and *C*_min_ are obtained are given in Sections “Optimal setting parameters for influential neighbors identification” and “Parameter selection” in Material and Methods.

**Fig 5:**
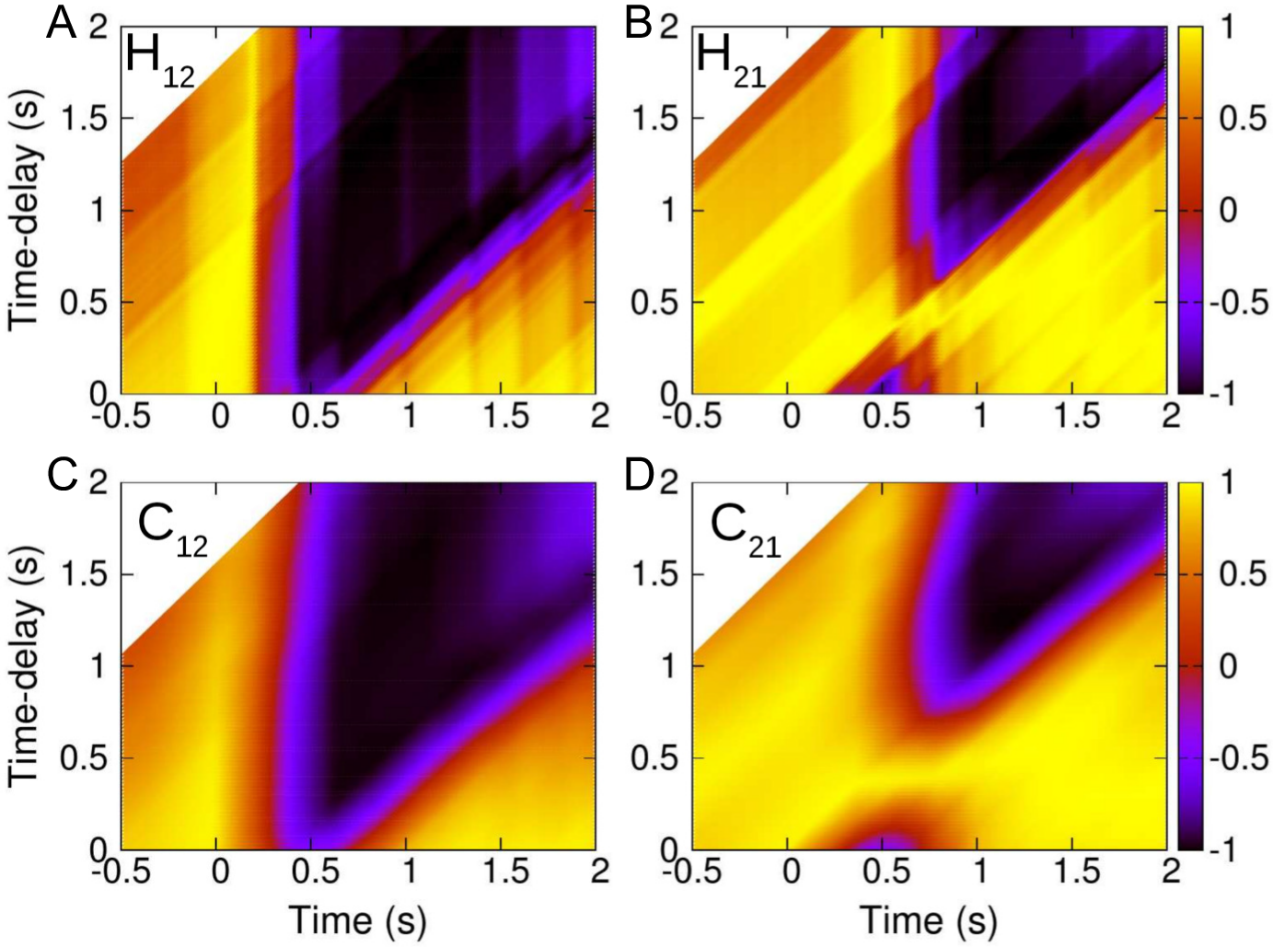
Directional correlations between fish *F*_1_ and *F*_2_. (A) Directional correlations *H*_12_(*t*, *τ*) and (B) *H*_21_(*t*, *τ*) for *t* ∈ [−0.5, 2] and *τ* ∈ [0, 2] and their corresponding average over a time-window of width 2*w* = 0.4 s: (C) *C*_12_(*t*, *τ*, *w*), (D) *C*_21_(*t*, *τ*, *w*). Yellow regions: *H*_*ij*_ ≈ 1 and *C*_*ij*_ ≈ 1, *i.e.*, fish have the same direction with a time-delay *τ*. Dark regions: *H*_*ij*_ ≈ −1 and *C*_*ij*_ ≈ −1, *i.e.*, fish have opposite directions. The white upper-left corners indicate the *τ* is larger than the minimal time considered in this data set.

Fig 5 shows the directional correlation *H*_12_ and its time-average *C*_12_ between fish *F*_1_ and *F*_2_ along the collective U-turn depicted in Fig 3. Left (resp. right) panels aim to indicate the alignment of fish *F*_1_ (resp. *F*_2_) at each time *t* with respect to the alignment of fish *F*_2_ (resp. *F*_1_) at an earlier time *t* − *τ*. Panels A and C show respectively that for all *τ*, there is always an interval of time during which *H*_12_(*t*, *τ*) ≈ −1 and *C*_12_(*t*, *τ*) ≈ −1 (dark region), meaning that for all time-delays there is always an interval of time in which fish have opposite directions. Moreover, the larger the time-delay, the wider the black region where the direction of *F*_1_ is opposite to the direction of *F*_2_ at the previous time.

On the other hand, the figures of the directional correlation of *F*_2_ with *F*_1_, especially Panel D, show a connected region in which the correlation *C*_21_(*t*, *τ*) remains positive and above the threshold (yellow in the figure) around *τ* ≈ 0.42 s where *H*_21_ ≈ 1 during all the time interval [−0.5, 2 s]. This strongly suggests that, during this time interval, *F*_2_ is copying the behavior of *F*_1_ with a 0.42 s time-delay, denoted *τ*_2,1_ for this specific U-turn. Thus, one can consider that *F*_1_ is influencing *F*_2_ with time-delay *τ*_2,1_, while *F*_2_ is not influencing *F*_1_ in this specific case. This influence dynamics is illustrated in Fig 3D by drawing an arrow at time *t* from *F*_*j*_ to *F*_*i*_ when *F*_*j*_ satisfies the condition *C*_*ij*_(*t*, *τ*, *w*) > *C*_min_ for being an influential neighbor of *F*_*i*_ at time *t*, which in turn receives this influence and responds by copying the exhibited heading with a time-delay *τ*.

**Fig 6:**
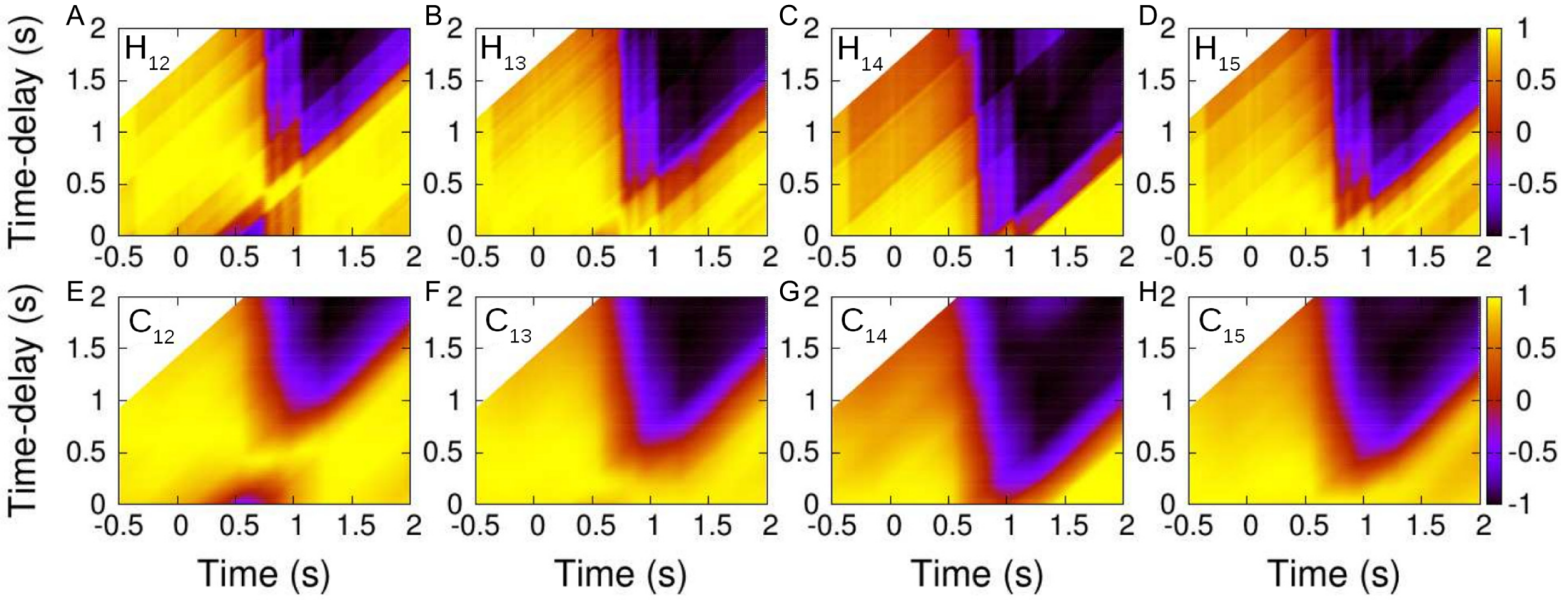
Directional correlation of fish *F*_1_ with the other fish *F*_*j*_, *j* = 2, …, 5. Directional correlations *H*_1*j*_(*t*, *τ*) (panels ABCD) for *t* ∈ [−0.5, 2] and *τ* ∈ [0, 2], and their corresponding average *C*_1*j*_(*t*, *τ*) (panels EFGH) over a time-window of width 2*w* = 0.4 s. Yellow regions: *H*_*ij*_ ≈ 1 and *C*_*ij*_ ≈ 1 (fish have the same direction with a time-delay *τ*). Dark regions: *H*_*ij*_ ≈ −1 and *C*_*ij*_ ≈ −1 (fish have opposite directions). In the upper-left corners the white color indicates that *τ* is larger than the minimal time considered in this data set.

Using the same procedure for the *N* = 5 case depicted in Fig 4, we draw Fig 6 that shows *F*_1_ copying *F*_2_ with a time-delay *τ*_1,2_ ≈ 0.5 s (Panels A and E). *F*_1_ also copies *F*_3_ and *F*_5_ with, respectively, *τ*_1,3_ ≈ 0.2 s (Panels B and F) and *τ*_1,5_ ≈ 0.1 s (Panels D and H), but it doesn’t copy *F*_4_ (Panels C and G). The influential neighbors of *F*_1_ are thus *F*_2_, *F*_3_ and *F*_5_, at different times and with different time-delays. We have calculated the rest of the correlations for all pairs of fish (see S1 Fig for an overview of all the heading correlations). As for the *N* = 2 case, these relations are illustrated by arrows going from the influential neighbors to the reacting fish in Fig 4D.

### Effect of bursting on the transmission of information

The specific behavior of *H. rhodostomus*, namely, the successive alternation of bursts and coasts [15], leads us to ask whether these abrupt changes of acceleration and speed can provide information that other fish could use to adjust their own movement. To address this aspect we study whether there is any correlation between the bursting activity of one fish at time *t* and the fact that this fish is an influential neighbor of another fish shortly after time *t*.

A burst corresponds to a brief phase of acceleration during which most changes in fish heading occur [15]. Panels E in Figs 3 and 4 show the bursting activity of each fish *F*_*i*_, *i* = 1, …, *N*, and that of its influential neighbors. For each fish *F*_*i*_, we draw a dot at time *t* and ordinate *i* if fish *F*_*i*_ is displaying a burst precisely at time *t*. Dot color at ordinate *i* corresponds to fish *F*_*i*_’s color. The absence of a dot at a given time denotes that the fish is in a coasting phase at that time.

A second row of colored dots is drawn at ordinate *i* − 0.5 for some values of *t* when two conditions are met: (1) Fish *F*_*i*_ is being influenced at those times by one or more fish *F*_*j*_, *j* ∈ {1, …, *N*}, *j* ≠ *i*, whose identity is given by the color of the dots, and (2) the influential fish *F*_*j*_ was bursting when it was influencing *F*_*i*_ at time *t* − *τ* earlier. If *F*_*i*_ has more than one influential neighbor at time *t*, the dot drawn at time *t* in row *i* − 0.5 has the color of the *F*_*j*_ fish with the highest index *j*.

In Fig 3E, red dots at *i* = 1 mean that fish *F*_1_ is bursting at those time-steps and coasting at the other time-steps, and red dots at *i* − 0.5 = 1.5 indicate that, first, *F*_1_ is the influential fish of *F*_2_ at those time-steps, and second, *F*_1_ was bursting when it was earlier influencing *F*_2_. In turn, there are two possible reasons to explain the absence of red dots at *i* − 0.5 = 1.5 for certain time values: either *F*_2_ has no influential neighbor, or *F*_1_ was coasting. To assess which of the two explanations is valid, one needs to look at Fig 3D. For example, the absence of dots at *i* − 0.5 = 1.5 during 0.57 s and 0.62 s is due to *F*_2_ having no influential neighbors, while the absence of dots in the same row between 0.75 s and 0.81 s results from the fact that *F*_1_, which is the influential neighbor of *F*_2_, is in a coasting phase at time *t* − *τ* (in this example the delay was found to be *τ* = 0.42 s).

Fig 3E shows that the bursting activities of both the focal fish and its influential neighbor are not directly correlated, suggesting that the primary source of information for fish to adjust their movements is the distance, orientation and angular position of their neighbors [15]. The same conclusion is obtained for *N* = 5. By focusing on fish *F*_2_ for example, Fig 4E shows that there is no systematic overlap between the yellow dots at *i* = 2 and those at *i* − 0.5 for *i* ≠ 2, suggesting that the correlation between the bursting activity of a fish and that of their influential neighbors is marginal.

### Number of influential neighbors

For all U-turns, we have counted the number of frames in which a fish is an influential neighbor, that is, the number of frames where the above described condition for identifying influential neighbors is met. When there are only two fish, a fish is found to be the influential neighbor 30% of the time spent in a U-turn. In groups of five fish, this proportion grows up to 62%.

**Fig 7:**
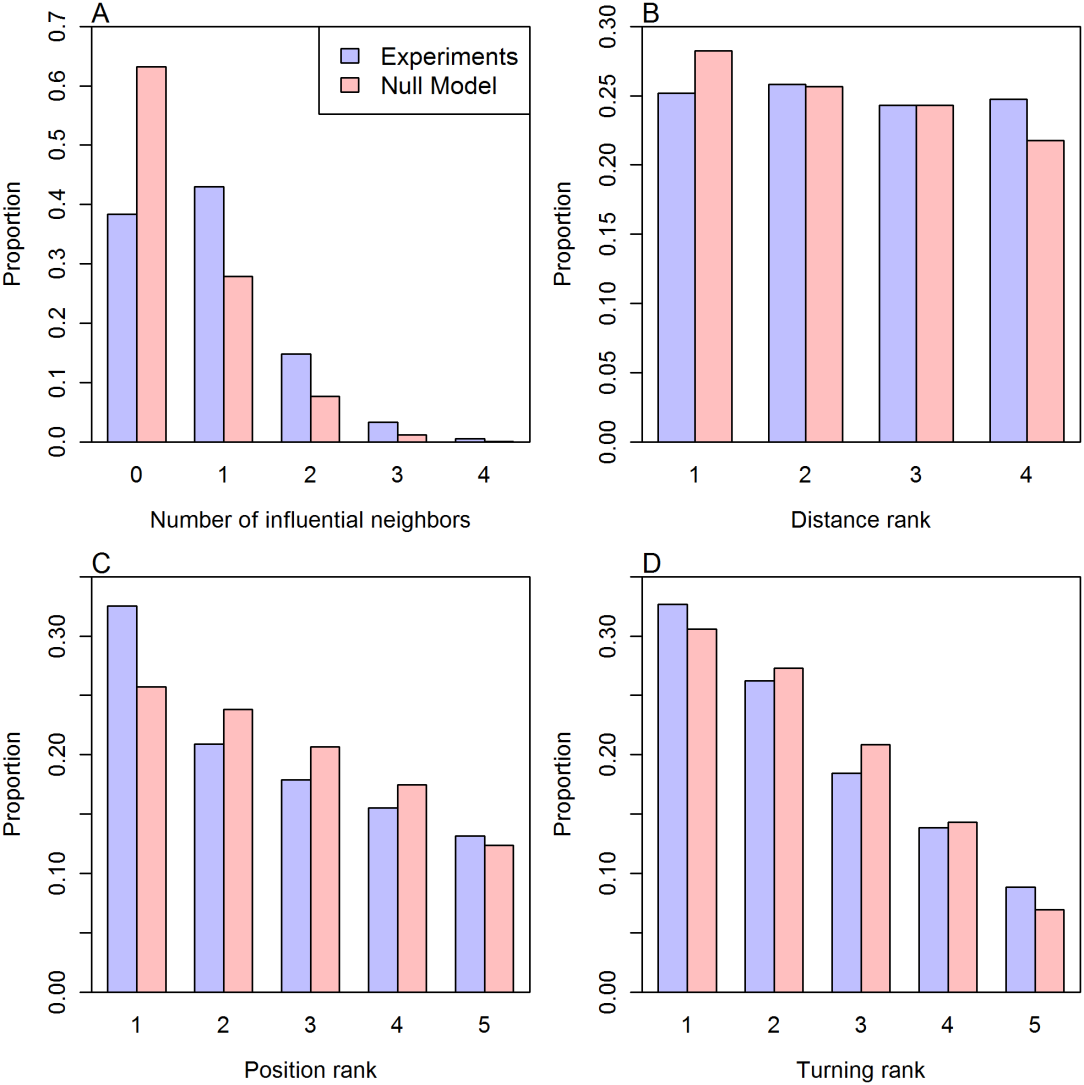
Number, location and temporal occurrence of influential neighbors. Cumulative analysis of collective U-turns of over 475 experimental (blue) and 1000 artificial (red) observations in groups of *N* = 5 fish. (A) Number of influential neighbors.(B) Distance rank of influential neighbors with respect to the focal fish. (C) Position rank of influential neighbors in the group. (D) Turning rank of influential neighbors. Histograms represent the proportion of time during which influential neighbors have been observed in a given class. The procedure to construct the artificial observations is presented later and in the section “Null model” in Materials and Methods.

We have counted the number of influential neighbors *N*_if_ a fish *F*_*i*_ has during a U-turn in groups of five fish, finding that in most cases, a fish has only one or two influential neighbors (for 58% of the time spent in a U-turn *N*_if_ = 1 or 2); see Fig 7A. The most frequent case is *N*_if_ = 1 (43%). Having more than one influential neighbor is frequent (19%), but less than having no influential neighbors (38%). The cases where there are more than two influential neighbors are negligible (less than 4% of the total time spent in U-turns).

For each fish *F*_*i*_, we have calculated the respective distance *d*_*ij*_(*t*) at which the other *N* − 1 fish *F*_*j*_ are from *F*_*i*_ during the U-turns, thus establishing a rank order among the neighbors influenced by *F*_*i*_. We have then compared the influence of close neighbors with those of distant neighbors, finding no correlation between the distance rank of a neighbor and the influence it exerts on the focal fish. This is shown in Fig 7B, where we have depicted the distribution of the distance rank of influential neighbors with respect to a focal fish. The figure shows that fish spent the same proportion of time (≈ 25%) being an influential neighbor of a focal fish independently of their distance rank. In other words, influential neighbors are not necessarily the closest ones.

When trying to identify events of causal influence by means of correlations, it is crucial to keep in mind that correlation does not imply causation. We thus have controlled the effects of potential chains of influence, where *e.g.* fish *F*_1_ is highly correlated with *F*_3_ not because *F*_1_ is directly influencing *F*_3_, but because *F*_1_ is influencing fish *F*_2_, which in turn is influencing *F*_3_. To check the impact of these chains of influence on our results, we have removed from our data all the pairwise influence data that correspond to the following situation: if *F*_1_ is influenced by both *F*_2_ and *F*_3_ and *F*_2_ is simultaneously influenced by *F*_3_ (or *F*_3_ is influenced by *F*_2_), then we removed the pairwise correlation (focal fish, influential neighbor) corresponding to (*F*_1_, *F*_2_) (or (*F*_1_, *F*_3_)). After removing 7172 out of 69703 data points and recomputing the results with the remaining data, we found that our results remain practically unchanged.

We have also calculated the position rank that each fish occupies in the group during a collective U-turn, finding that influential neighbors are mostly located in the front region of the group: 32% in the leading most advanced position, and 20% in the second place; see Fig 7C. Noticeably, influential neighbors can be found in the back of the group (in 29% of the cases in the fourth or fifth position), and even in the last position (a non-negligible 13% of cases).

We also paid attention to the order in which each fish starts its individual U-turn during a collective U-turn, finding that influential neighbors are those that most frequently turn earlier (32% of the cases), and that this relation decreases linearly; see Fig 7D. It is again noticeable that influential fish can be found to be the last turning fish (in 8% of the cases).

The apparently surprising fact that influential fish can be found in the back of the group and that the last fish turning can be an influential fish is due to the anisotropic perception of fish and their relative orientations during U-turns. But these findings have to be understood in the light of our specific time-dependent characterization of influential neighbor. If, for instance, *F*_1_ turns first and influences *F*_2_, *F*_2_ will turn with some time-delay after *F*_1_. Then, when *F*_2_ is at half of its individual turning process, *F*_2_ can be rotating in the same direction as *F*_1_ in such a way that *F*_1_, influenced by *F*_2_, slightly adjusts its direction. We would then say that *F*_2_, which is the last turning fish, has influenced *F*_1_, the first turning fish.

In order to compare different collective U-turns, we define a normalized time $\bar{t}=\left(t-t_{s}\right)/\left(t_{e}-t_{s}\right)$ in terms of the actual time *t* and the starting and ending time of each U-turn, so that the duration of a U-turn is now $\bar{t}=1$. Thus, $\bar{t}=-1$ corresponds to a time as long as the U-turn duration previous to the start of the U-turn, and $\bar{t}=2$ corresponds to a time as long as the U-turn duration after the end of the U-turn. We have calculated the instantaneous value of the average speed $V\left(t\right)=\langle ∥\vec{v}\left(t\right)∥\rangle$, the average group polarization P(t)=〈P(t)〉 and the average number of influential neighbors $N\left(t\right)=\langle N_{\text{if}}\left(t\right)\rangle$. Here, angle brackets refer to the average across all fish in the U-turn along a time-window containing the collective U-turn.

**Fig 8:**
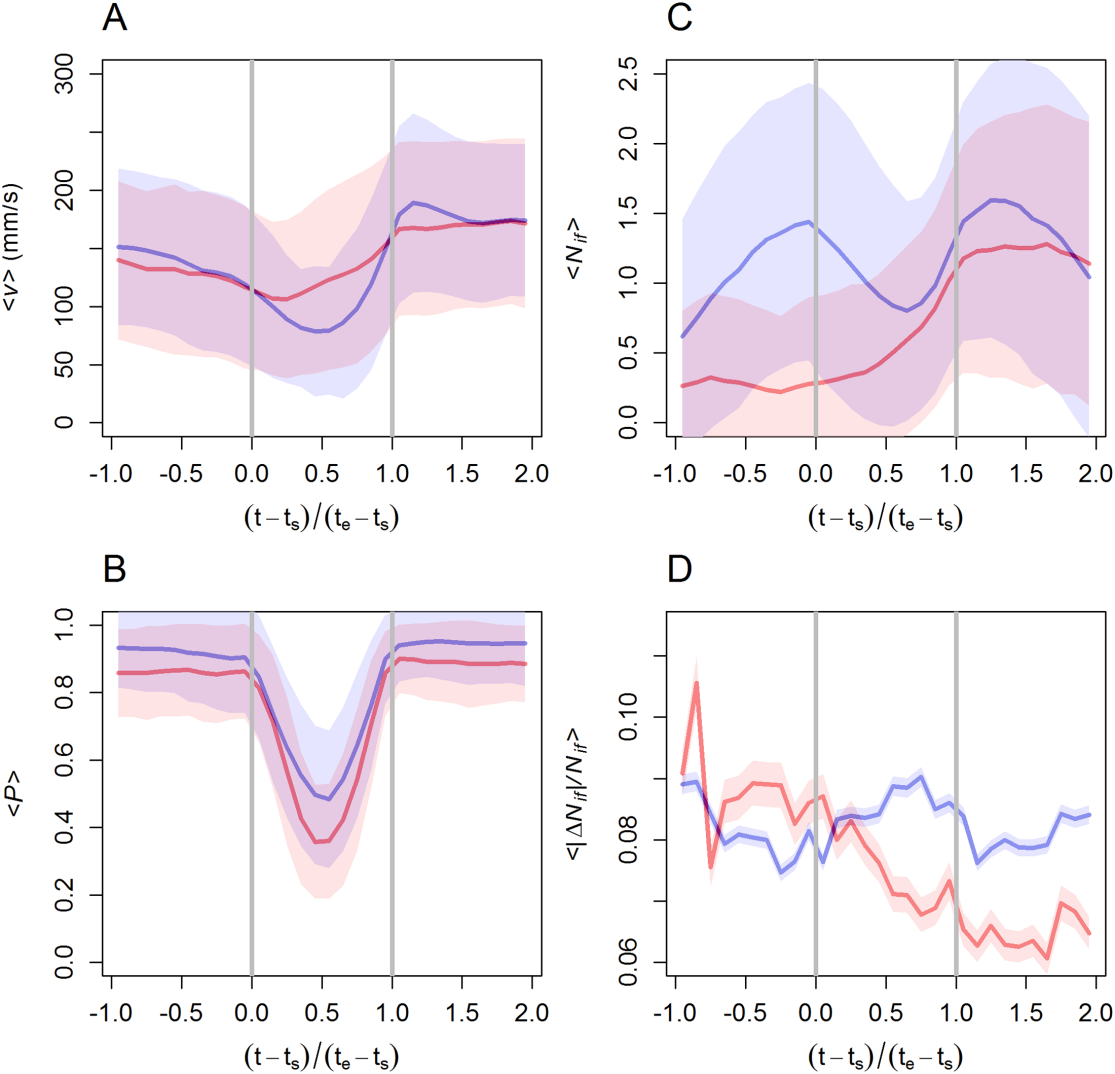
U-turn dynamics in groups of *N* = 5 fish. We depict here the temporal dynamics for the average velocity, average polarization, number of influential neighbors and its variation in over 475 experimental (blue) and 1000 artificial (red) recordings of collective U-turns. (A) Average speed V(t). (B) Average group polarization P(t). (C) Average number of influential neighbor N(t) per focal fish. (D) Average of the absolute variation in the number of influential neighbors |Δ*N*_if_| divided by the number of influential neighbors *N*_if_, defined in Eq (5): 〈*η*(*t*)〉. Horizontal axis denotes normalized time $\bar{t}$, where *t*_*s*_ and *t*_*e*_ denote the start and end of the collective U-turn respectively. The procedure to construct the artificial observations is presented later and in the section “Null model” in Materials and Methods.

Fig 8A and 8B show respectively the time evolution of V(t) and P(t) during the collective U-turns in groups of 5 fish. The description of the specific U-turn presented in Fig 4 is also valid for the general case: the speed decreases before the U-turn (from V(-1)≈150 mm/s to V(0)≈115 mm/s), it reaches a minimum at half the U-turn duration $\bar{t}=0.5$ (V(0.5)≈70 mm/s), and it then grows to a higher value after the U-turn (V(1.5)≈165 mm/s). A very similar behavior was found in groups of 2, 4, 8 and 10 fish of the same species in [28]. At the same time, the polarization is very high and almost constant outside the U-turn ($P(\bar{t})\approx 0.95$), and exhibits a perfect V-shape during the U-turn, with the high values ($P(\bar{t}=\left\{0,1\right\})\approx 0.93$) reached at exactly the instants where the start and end of the U-turn takes place $\bar{t}=0$ and $\bar{t}=1$, and the minimum value (P(0.5)≈0.48) at the middle of the U-turn. As expected, the average group polarization $P(\bar{t})$ significantly decreases during the U-turn to almost half the value it has outside the U-turn. Right after reaching this minimum, there is a sharp increase of speed and polarization as more fish adopt the new direction of motion.

Fig 8C shows that before the U-turn the average number of influential neighbors N(t) increases until a maximum value is reached right before the start of the U-turn (N(-0.1)≈1.45). During more than one half of the U-turn, N(t) decreases until a minimum (N(0.6)≈0.8), and grows again beyond the end of the U-turn until a second maximum (N(1.2)≈1.6, twice the height of the minimum). After that, all fish have completed their U-turns and N(t) decreases again.

When the polarization is very high, the time-delay with which influential neighbors are detected is often too small in comparison with biologically realistic reaction times *τ*_R_, so that these influential neighbors are not taken into account (we used *τ*_R_ = 0.04 s; see Section “Optimal setting parameters for influential neighbors identification” in Materials and methods). This is the reason why the average number of influential neighbors N(t) appears to be smaller in regions outside the U-turn, than when the U-turn is just about to start ($\bar{t}\approx -0.1)$ or slightly after its end ($\bar{t}\approx 1.2$). Meanwhile, the decrease of N(t) in the middle of the U-turn has a different origin: once a fish has started to turn around, there is no real need of updating its alignment according to all its neighbors. That fish can safely reverse its motion by keeping the alignment with only one of those neighbors and even not paying attention to them for some period of time.

Another indicator of how fish make decisions while turning is how frequently a focal fish pays attention to other individuals. We define the relative variation of the number of influential neighbor per fish *N*_if_(*t*) between two successive time-steps as follows:

$$\begin{array}{c} \eta \left(t\right)=\frac{\left|N_{\text{if}}\left(t+\Delta t\right)-N_{\text{if}}\left(t\right)\right|}{N_{\text{if}}\left(t\right)}, \end{array}$$ (5)

denoting by Δ*t* the time-step between frames (Δ*t* = 0.02 s).

We have depicted the time-evolution of the average 〈*η*(*t*)〉 in Fig 8D, finding that 〈*η*(*t*)〉 remains essentially constant before, during and after the U-turn event, the amplitude of its variation being smaller than 10% of the signal (0.007 and 0.08, respectively).

Since the average number of influential neighbors N(t) is smaller when fish are engaged in the U-turn than right before or right after the U-turn, a constant average 〈*η*(*t*)〉 suggests that fish adjust their heading more frequently during the U-turn than outside the U-turn. Indeed, in the middle of a U-turn, no real common direction of motion exists (P(t)≈0.5), that is, there is a high diversity of headings, so that fish have to frequently update their direction by paying attention to different neighbors.

### Spatial organization of influential neighbors

We are now interested in determining the dynamical spatial organization of the influential neighbors of a focal fish. The relative state of a fish *F*_*j*_ with respect to a focal fish *F*_*i*_ is characterized by several parameters: the relative position of the neighbor ${\vec{u}}_{ij}={\vec{u}}_{j}-{\vec{u}}_{i}$, where ${\vec{u}}_{i}$ is the vector position of *F*_*i*_ in cartesian coordinates, the distance between them $d_{ij}=∥{\vec{u}}_{ij}∥$, the viewing angle of *F*_*j*_ relative to the direction of *F*_*i*_ [26], which is the angle *θ*_*ij*_ with which *F*_*i*_ perceives *F*_*j*_ (note that *θ*_*ij*_ is not necessarily equal to *θ*_*ji*_), the relative velocity ${\vec{v}}_{ij}={\vec{v}}_{j}-{\vec{v}}_{i}$, and the relative heading *ϕ*_*ij*_ = *ϕ*_*j*_ − *ϕ*_*i*_. All these quantities are time-dependent. We have calculated their average value for all the U-turns in a uniform spatial grid of square cells to facilitate the interpretation of the vector field of these continuous variables. Each square cell, of side 20 mm, shows the average of the arbitrarily different number of values contained in the cell.

**Fig 9:**
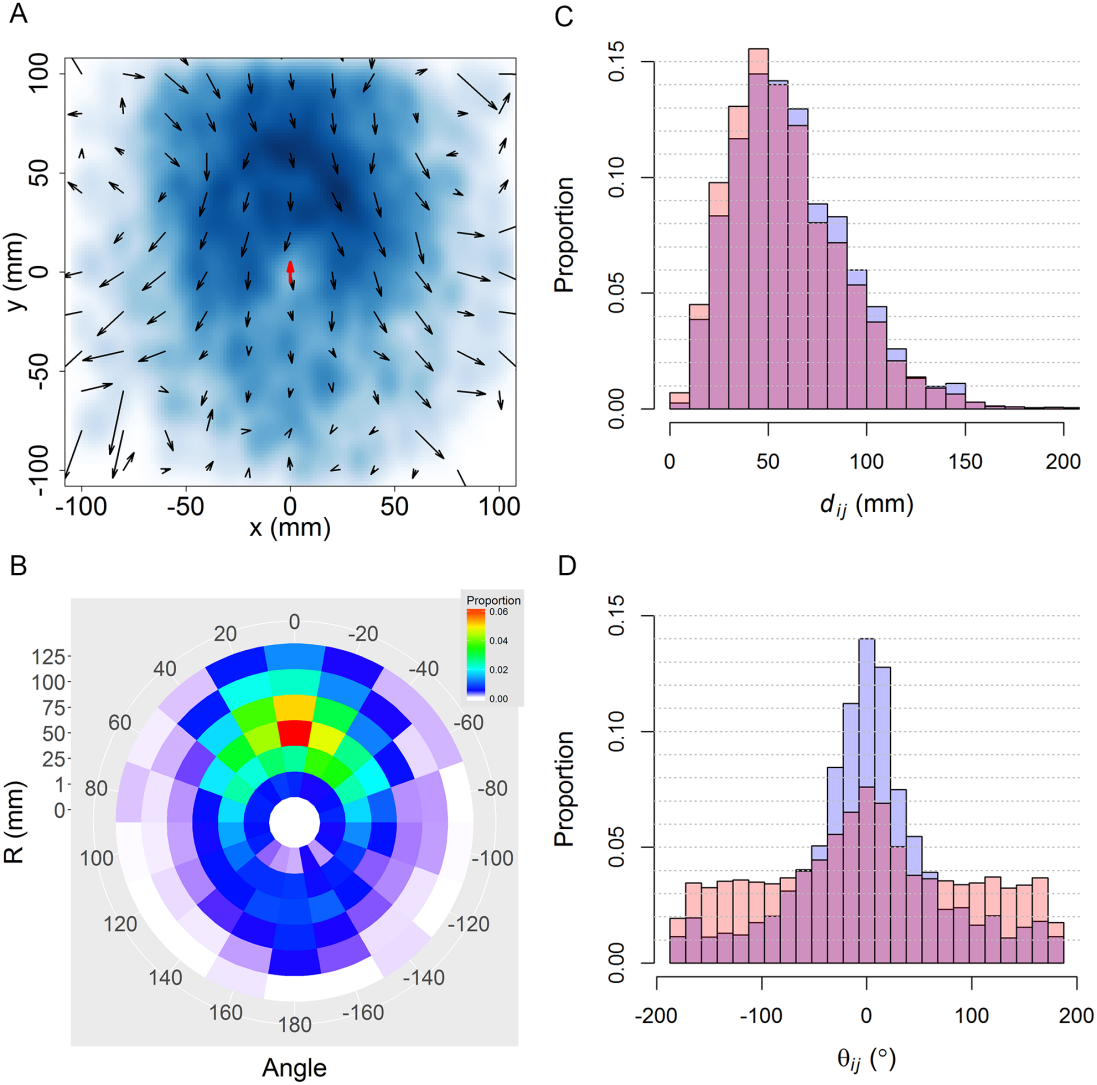
Spatial and velocity distributions of influential neighbors around a focal fish in groups of 2 individuals. (A) Density map of influential neighbors’ location (blue) and their average relative velocity field (arrows) with respect to the focal fish (red arrow). (B) Average spatial distribution of influential neighbors in polar coordinates (red: highest frequency; dark blue: low frequency; white: frequency equals zero). (C) and (D): Distributions of the distance *d*_*ij*_ and the angle of exposure *θ*_*ij*_ respectively. Blue histograms: *F*_*j*_ is an influential neighbor of *F*_*i*_; orange: *F*_*j*_ is a neighbor of *F*_*i*_, not necessarily influencing *F*_*i*_; dark pink: overlap between the two.

Fig 9A shows the density map of the relative position of the influential neighbor with respect to the focal fish when *N* = 2. The intensity of color is proportional to the frequency of occupation of the grid cell, showing that the influential neighbor is mostly located in front of the focal fish and at a distance of one to three body lengths from the focal fish. The same information is quantified in Panel B with a heat map in polar coordinates, highlighting the most frequent location of the influential neighbor.

The average relative velocity $\langle {\vec{v}}_{ij}\rangle$ is shown in Fig 9A (arrows), superimposed to the density map. The vector field shows that when the influential neighbor is in front of or behind the focal fish (sin〈*θ*_*ij*_〉 ≈ 0), both fish move at similar speed although the focal fish is a little bit faster (the small black arrows are pointing in the opposite direction to the red one) and the difference in heading is also small. However, when the influential neighbor is on the sides of the focal fish, relative speed and heading difference tend to vary more as the distance between them increases.

The distributions of distances *d*_*ij*_ and exposure angles *θ*_*ij*_ between a focal fish and its neighbors are depicted in Panels C and D of Fig 9 respectively. We find, on the one hand, that their most frequent separation is 62.6 mm ± 29.7 mm (mean and standard deviation of histogram in Fig 9C), a value that is consistent with previous results where it was shown that the behavioral reactions of a fish depend on the angular position of its neighbors, as a consequence of the anisotropic perception of the environment [15].

On the other hand, the distribution of the exposure angle of fish *F*_*j*_ to the focal fish *F*_*i*_ is narrower when *F*_*j*_ is influencing *F*_*i*_ than when *F*_*j*_ is a neighbor of *F*_*i*_, not necessarily influencing *F*_*i*_. As both distributions are centered on *θ*_*ij*_ = 0, this shows that *F*_*j*_ is more frequently located in front of *F*_*i*_ when *F*_*j*_ is an influential neighbor of *F*_*i*_ than in the case when *F*_*j*_ is just a neighbor of *F*_*i*_.

**Fig 10:**
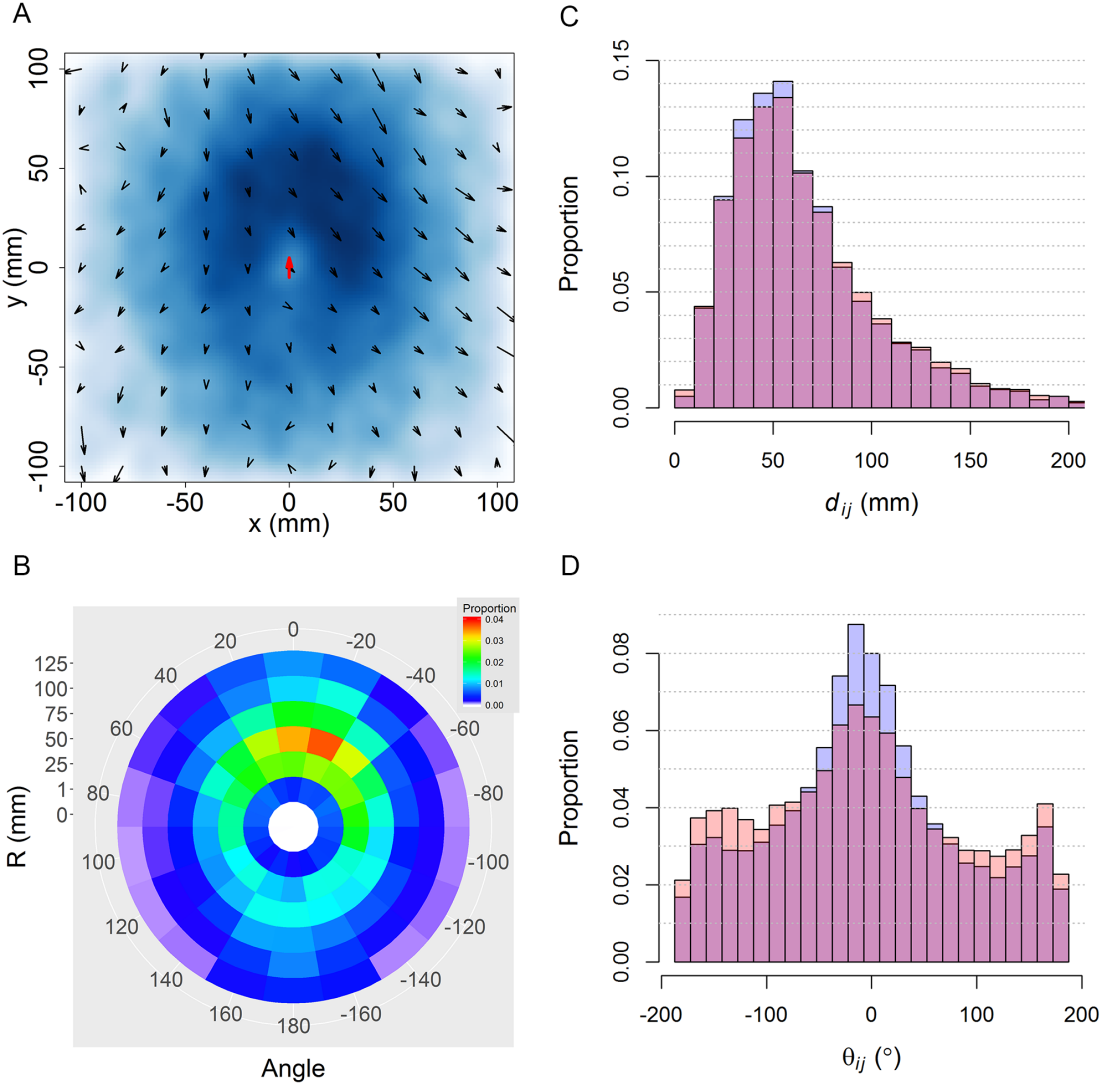
Spatial and velocity distribution of influential neighbors around a focal fish in groups of 5 individuals. (A) Density map of influential neighbors’ location (blue) and their average relative velocity field (arrows) with respect to the focal fish (red arrow). (B) Average spatial distribution of influential neighbors in polar coordinates (red: highest frequency; dark blue: low frequency; white: frequency equals zero). (C) and (D): Distributions of the distance *d*_*ij*_ and the angle of exposure *θ*_*ij*_ respectively. Blue histograms: *F*_*j*_ is an influential neighbor of *F*_*i*_; orange: *F*_*j*_ is a neighbor of *F*_*i*_, not necessarily influencing *F*_*i*_; dark pink: overlap between the two.

Fig 10 shows similar results for groups of *N* = 5 fish. Influential neighbors are more frequently located in front of the focal fish (although with a slight shift to the right; see Panels A and B) and at a mean distance of 67.5 mm ± 40.6 mm (Panel C).

In turn, the velocity field has a smaller intensity and is much more homogeneous than in the case where *N* = 2. A slight asymmetry can also be observed (not noticed when *N* = 2) with fish located in front and slightly to the right of the focal fish having a higher velocity than those located elsewhere. Moreover, the distribution of exposure angles is more dispersed than in the case of two fish, meaning that influential neighbors are exposed to the focal fish with a larger diversity of angles, something that is simply due to the higher number of fish.

The difference in the homogeneity of the velocity field between groups of 5 and 2 individuals is not necessarily the result of averaging over a larger number of individuals. Although averaging over fish data pairs may reduce the uncertainty in the extracted parameter values, it is well-known that the level of homogeneity in the direction of motion of the school increases with group size [29]. But one also ought to consider that specific values of delay and curvature the individuals adopt during the U-turns could help to limit variability in coordinating the group. Some theoretical studies support this idea: simplified models of velocity alignment with additive noise have shown semi-analytically the existence of delay and rate of turn values that minimise the fluctuations in the variance of the individual speed [30], and flocking models of self-propelled particles have also shown that delay can be tuned to increase stability and alignment of the group [31].

**Fig 11:**
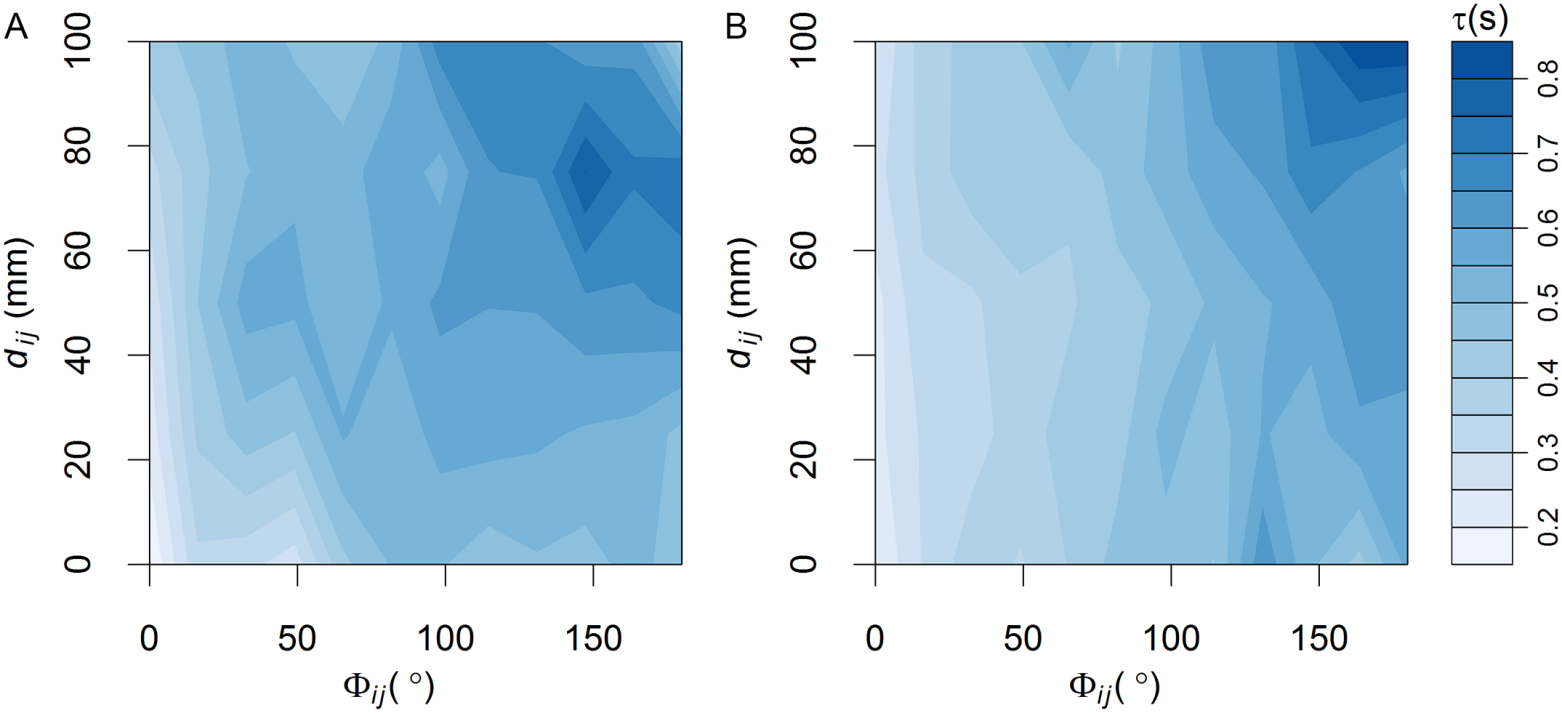
Time-delay dependence on heading difference and separation distance. Time delay *τ* extracted from the empirical observations as a function of heading difference *ϕ*_*ij*_ and separation distance *d*_*ij*_. (A) *N* = 2, (B) *N* = 5. In both cases, the larger the heading difference and the distance, the longer the time-delay.

Finally, we have analyzed the variation of the time-delay *τ* as a function of both the distance between the focal fish and its influential neighbors *d*_*ij*_ and the difference of heading *ϕ*_*ij*_, finding that in both cases *N* = 2 and *N* = 5, the time-delay increases with respect to both the distance *d*_*ij*_ and the heading difference *ϕ*_*ij*_ (see Fig 11). This result can be understood because during a U-turn the fish speed is decreasing and two fish can display larger reaction times the more separated they are and the less aligned they are.

### A null model to detect spurious correlations

As already mentioned in the introduction, establishing causal influence on the basis of correlation measures requires controlling for spurious effects. Although our experimental data correspond to a specific collective behavior in which individuals influence each other, the relatively short time-windows over which cross-correlation are averaged and the use of several parameters through sensitivity analysis can weaken the accuracy of our results. To demonstrate that the particular detections of influential neighbors are not purely due to chance, we generated random artificial U-turns events by bootstrapping the data and applying the same procedure used to analyze collective U-turns in our experiments.

The null model is built for groups of 5 fish, for which our experimental data provide *M* = 2375 individual trajectories (5 × 475 collective U-turns). For every fish *F*_*i*_, *i* = 1, …, *M*, the trajectory is rotated so that the individual turning point of the fish (where sin(*θ*_*wi*_) = 0) is located in the upper part of the tank, by randomly sampling the new angular position *ψ*_*i*_ in the interval [*π*/2 − *ξ*, *π*/2 + *ξ*], where *ξ* is a small angle (we used *ξ* = *π*/12). Similarly, the time scale of each fish is shifted by sampling the instant of turning in the time interval [−*ζ*, *ζ*], where *ζ* is a short time (we have used *ζ* = 1 s). Then, five trajectories are randomly sampled, each one from a different randomly sampled collective U-turn, and mirrored if necessary so that the five individual U-turns are done in the same direction, clockwise or anti-clockwise. This way, the five fish of the artificial U-turn make their individual U-turn approximately at the same place and approximately the same time. For more details, see the section “Null model” in Materials and methods.

We have produced 1000 artificial collective U-turns; S9 Fig shows a collection of 10 of them. The results of our analysis are shown in red in Figs 7 and 8. As expected, they reveal clear differences between artificial and experimental U-turns.

Fig 7A shows that in artificial U-turns the proportion of time during which a focal fish has no influential neighbor is more than 63% of the time, while in the experiments it was less than 39%. The analysis also reveals that in artificial U-turns a focal fish has one influential neighbor for less than 28% of the time, while in the experiments, the proportion raises to 43%. Similarly, Fig 8C shows that the average number of influential neighbors $N\left(t\right)=\langle N_{\text{if}}\left(t\right)\rangle$ is much smaller in artificial U-turns (≈ 0.4) than in real U-turns, where N(t) is almost always greater than 1. Note that the increase of N(t) during U-turns in artificial data is the consequence of the channeled motion of fish by the corridor. Moreover, the variation of N(t) along time, including the transients preceding and following the U-turn, decreases in artificial U-turns while it remains constant and with a higher value in experiments.

Fig 7B shows that distance rank has no significant effect on which fish is the influential one, both in experiments and in artificial U-turns. The decreasing number of influential neighbors comes from the fact that the tank is circular and the method we use. If the tunnel had been a straight corridor, we should have detected no decrease in our null model. However, in a circular tank, because of the geometrical constraints imposed by the curvature, even when two fish are both swimming in the same direction (*i.e.*, clockwise or anti-clockwise), as the distance between fish increases, our method will detect a decrease of correlation. While Fig 7C confirms that influential neighbors are slightly more often ranked in the first position of the group, this effect is much more pronounced in the experiments. In fact, Figs 7B, 7C and 7D and 8A and 8B show that the selected null model satisfactorily reproduces the typical spatiotemporal behavioral patterns of real U-turns: the position and turning ranks are almost identical, as well as the variation of the average speed and the average group polarization, although the V-shape of the average polarization in real U-turns is significantly sharper than in artificial U-turns.

An additional, albeit expected, result of our null model is the homogeneous (isotropic) spatial distribution of “influential neighbors”, while in real collective U-turns influential neighbors are mostly located in front of the focal fish; see S10A and S10B Fig, compared with Fig 10A and 10B.

## Discussion

By sharing information with other group members, schooling fish and other collectively moving animals can potentially improve their navigational accuracy (*e.g.* the many wrongs principle [32]), take better decisions (*e.g.* to avoid a predator [33]), or improve their abilities to sense the environment [34]. However, there are both physical and practical reasons why information is expected to be shared with only a few neighbors. Physical reasons involve material limitations, such as visual occlusions. Practical reasons often refer to trade-offs between sharing information, so that the group collectively selects a direction of motion, and deciding independently [35, 36].

Assuming that correlations between fish behavior rely to some extent on a causal influence, our analysis reveal that in groups of *H. rhodostomus*, during a collective U-turn, at any moment in time each fish only pays attention to a small number of neighbors whose identity regularly changes. We also find that the phases during which a focal fish is affected by one or two influential neighbors are interspersed with other phases during which its movement appears uninfluenced by the movement of neighbors. Moreover, influential fish are mostly located in front of the focal fish. The distance between a focal fish and its influential neighbors is about two body-lengths and the relative exposure angle is smaller than 60 degrees.

Our results bring insights on the way information on the neighborhood is processed by fish. Instead of having a synchronous update based on a fixed number of neighbors (topological neighborhood) or on all neighbors located within a fixed distance (metric neighborhood), our results suggest an asynchronous updating that does not depend on the distance between a focal fish and its influential neighbors. A similar asynchronous updating scheme has been previously introduced by Bode et al. [37] in a flocking model showing that it can give rise to emergent topological interactions consistent with the measures done on starling flocks [38].

It is however worth noting that our experiments, performed on small group sizes, may have prevented us from detecting any influence of the distance, since each of the four neighbors are located between one and three body lengths. In larger groups of fish moving in an unconstrained space, we expect the effective neighborhood of fish to result from the interplay between an asynchronous updating on a small number of neighbors and a modulation of the strength of interactions with the distance between fish [15].

Previous studies on the number and the spatial arrangement of influential neighbors led to different results depending on the species and on the procedure used to analyse the data. The work by Ballerini *et al*. [39] provides evidence that each bird within a starling flock (*Sturnus vulgaris*) coordinates its motion with a fixed number of closest neighbors, irrespective of their distance, while in mosquitofish (*Gambusia holbrooki*), one single nearest neighbor was sufficient to account for the large majority of the observed interaction responses [12]. In barred flagtails (*Kuhlia mugil*), it has been shown that different kinds of neighborhoods (Voronoi neighborhood and the *k* nearest neighbors (*k* ≈ 6 ∼ 8) were compatible with experimental data in a tank [13]. Our study points to a low number of influential neighbors. There are multiple possible explanations for the differences in the number of interacting neighbors found across the scientific literature. (*i*) It is possible that different animal groups interact with different numbers of neighbors. (*ii*) Temporal factors are also important [37], as interactions can be integrated in time to produce effectively larger neighborhoods. Here, we propose a third explanation (*iii*) based on the consideration that interaction responses such as attraction, alignment and avoidance are qualitatively different mechanisms that rely on different sensory-motor responses and, consequently, on different interacting neighborhoods. In particular, attraction and repulsion require to process information about the position of neighbors, while alignment is intrinsically a response dependent on orientation and velocity. These different interactions are likely to rely on different neural circuits (motion and form are typically processed by different brain areas in many animal groups [40, 41]) and hence might depend on different sets of influential neighbors: for instance, a focal individual could avoid collisions with its Voronoi neighbors, be attracted towards a different neighborhood of visually salient individuals and only process alignment information for one or two selected neighbors. It might also depend on different sets of influential neighbors: for instance a focal individual could avoid collisions with its Voronoi neighbors, be attracted towards a different neighborhood of visually salient individuals and only process alignment information for one or two selected neighbors.

It is thus natural to suggest that influential neighbors are intrinsically associated with different interaction mechanisms, which might also explain why fish point to different neighborhoods.

Our method for identifying influential neighbors is based on the computation of the time-dependent directional correlation between a focal fish and its neighbors. Of course, correlation does not imply causation, so that inferring causal influence between fish from directional correlation requires an extremely cautious methodology.

The methodology we proposed here is based on two solid procedural cornerstones. First, the data used in our study were carefully selected from a clearly recognizable behavior, the collective U-turns, where influence from neighbors undoubtedly exists, and thus should be, to some extent, responsible for a fundamental part of the correlations detected by our method. Time-delay between individuals’ direction choices has already been used to measure the interactions between group members in animal flocking. Specifically, Nagy *et al*. [23] used correlation delay times to reconstruct flight hierarchies in flocks of pigeons. Their approach consisted in integrating delay times over the entire trajectory to obtain a “leadership mark” for each individual. Our assumption is instead that the time-delay results from the individuals’ behavior and their environment, which varies in time depending on the information being gathered. To detect the response delay of each individual, we have instead followed the approach employed in [26] that allows for a change of delay over time. In fact, it is easy to show that the time delay between the same pair of fish is not constant, as revealed by our analysis of pair of fish (see Material and methods). Applying Nagy *et al*.’ method to different subsets of data in the same experiment, we found that the time delays between the same pair of fish vary substantially (see S2 Fig). The second methodological cornerstone is provided by the results of the null model that clearly show that the correlations we detected come from causal influence between neighbors and not from spurious random coincidences. The results of the null model also confirm that distance rank has no effect.

Identifying the number and position of influential neighbors is an essential step towards reconstructing behavioral cascades of information propagation across a group. Our method provides an accurate basis for mapping interaction network that does not rely on any assumption about the channel (*e.g.*, vision, sound or hydrodynamic interactions) mediating information transfer. We are confident that by adopting our technique to map interactions in different species and different experimental contexts we will gain a much more detailed understanding of the distributed information processing taking place in fish schools.

## Materials and methods

### Ethics statement

Our experiments have been approved by the Ethics Committee for Animal Experimentation of the Toulouse Research Federation in Biology *N*°1 and comply with the European legislation for animal welfare.

### Experimental procedures and data collection

**Fig 12:**
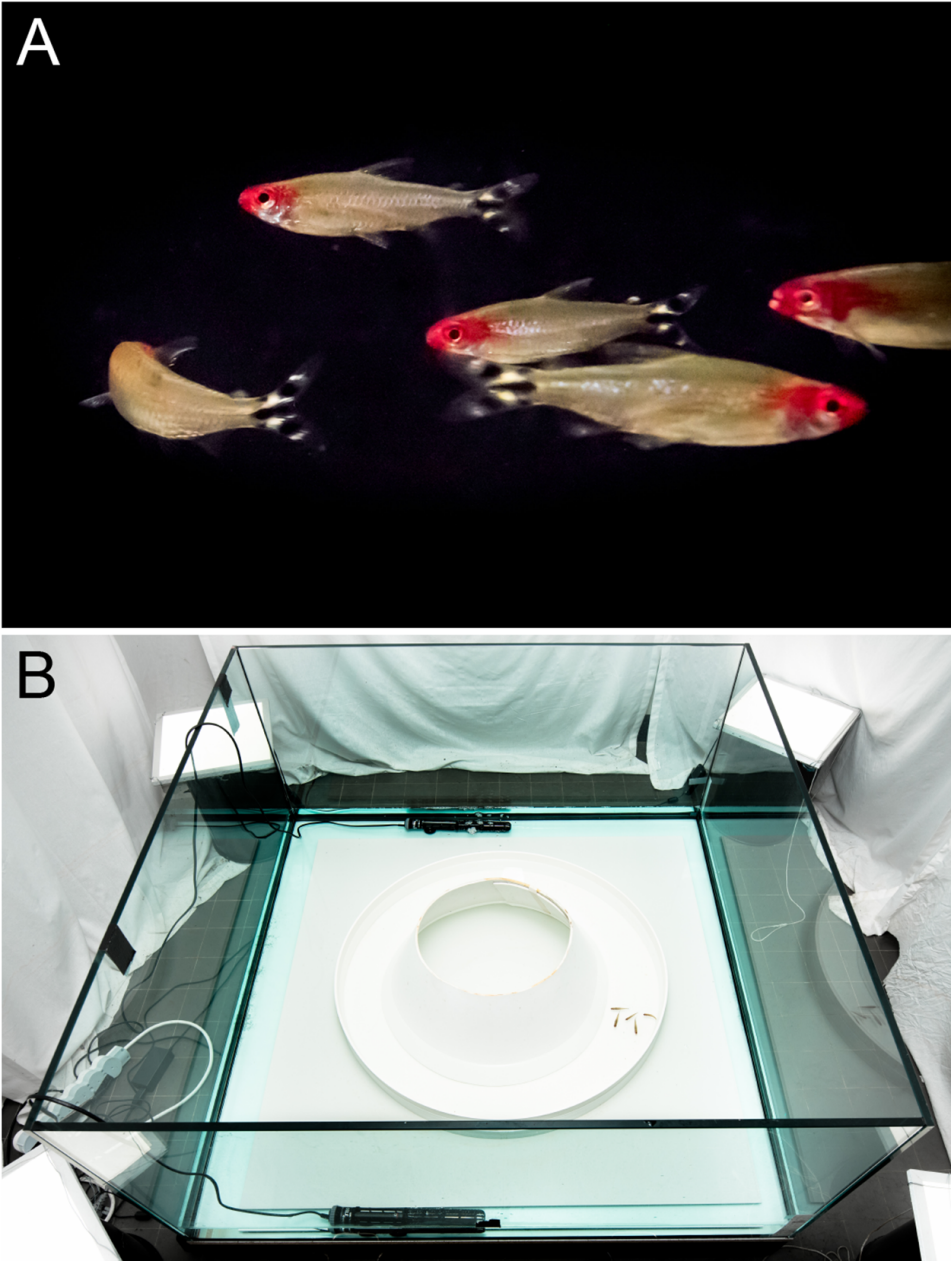
Fish and experimental setup. (A) A spontaneous U-turn initiated by a single fish in a group of five *Hemigrammus rhodostomus* fish. (B) Experimental ring-shaped tank, ©David Villa ScienceImage/CBI/CNRS, Toulouse.

*Hemigrammus rhodostomus* (rummy-nose tetras, Fig 12A) were purchased from Amazonie Labège (http://www.amazonie.com) in Toulouse, France. Fish were kept in 150 L aquariums on a 12:12 hour, dark:light photoperiod, at 27.7°C (±0.5°C) and were fed *ad libitum* with fish flakes. The average body length of the fish used in these experiments was 31 mm (± 2.5 mm). The experimental tank (120 × 120 cm) was made of glass and was set on top of a box to isolate fish from vibrations. The setup was placed in a chamber made by four opaque white curtains surrounded by four LED light panels to provide an isotropic lighting. A ring-shaped corridor was set inside the experimental tank filled with 7 cm of water of controlled quality (50% of water purified by reverse osmosis and 50% of water treated by activated carbon) heated at 28.1°C (±0.7°C) (Fig 12B). The corridor was made of a vertical circular outer wall of radius 35 cm and a circular inner wall with a conic shape of radius 25 cm at the bottom, so that the effective width of the corridor available to fish for swimming ranges from 10 cm at the bottom to 12 cm at the surface. The conic shape was chosen to avoid the occlusion on videos of fish swimming too close to the inner wall. Fish were randomly sampled from their breeding tank for a trial and were used at most in only one experiment per day. Groups of 2 or 5 fish were introduced in the experimental tank and acclimatized to their new environment for a period of 10 minutes. Their behavior was then recorded for one hour by a Sony HandyCam HD camera filming from above the setup at 50 images per second in HDTV resolution (1920x1080p). We performed 10 trials for each group size of 2 and 5 fish.

### Data extraction and pre-processing

The positions of each fish on each frame were tracked with idTracker 2.1 [10]. Fish were sometimes misidentified by the tracking software, for instance when two fish were swimming too close to each other for a long period of time. In those cases, the missing positions were corrected manually. All sequences with 50 consecutive missing positions or less were interpolated. Larger sequences of missing values were checked by eye to determine whether interpolating was reasonable or not; if not, namely the trajectory doesn’t look like a straight line, then merging positions with closest neighbors were considered. Time series of positions were converted from pixels into meters. The origin of the coordinate system was set to the center of the ring-shaped tank. Body orientation of fish were measured using the first axis of a principal component analysis of the fish shapes detected by idTracker 2.1.

### Detection and quantification of collective U-turns

Since the experiments were performed in an annular setup, the direction of rotation can be converted into a binary value: clockwise or anti-clockwise. We choose the anti-clockwise direction as the positive values for angular position. Before a U-turn event, all fish move in the same direction, say clockwise. Then, one fish, not necessarily the one located at the front of the group, changes its direction of motion to anti-clockwise direction. After a short transient, the other fish of the group display the same direction change, from clockwise to anti-clockwise. We defined the whole process of changing direction as a collective U-turn (see examples in Fig 1 and in S8 Fig). After data extraction and pre-processing, we found 1111 and 475 collective U-turns in groups of 2 and 5 fish, respectively. The duration distribution of collective U-turns in groups of 2 fish is shown in S3 Fig while the results for groups of 5 fish are shown in S4 Fig. Most of the collective U-turns last between 1 and 3 seconds, while the individual turning time usually lasts between 0.4 and 1 second.

The procedure used to define an individual U-turn for a fish *F*_*i*_ is as follows: we first determine the time *t*_*m*,*i*_ at which the sign of the angle of incidence of fish *F*_*i*_ changes sign (from negative to positive or vice versa). Then, starting from *t*_*m*,*i*_, we reverse time step by step until the first time at which the absolute value of the angle of incidence is higher than a threshold ${\bar{\theta }}_{s,i}$ is reached. We denote this time by *t*_*s*,*i*_. Similarly, we start again from *t*_*m*,*i*_ and go forward step by step until the first time at which the absolute value of the angle of incidence is higher than a second threshold ${\bar{\theta }}_{e,i}$ is reached. We denote this time by *t*_*e*,*i*_. To determine the values of the thresholds ${\bar{\theta }}_{s,i}$ and ${\bar{\theta }}_{e,i}$, we first compute the moving average of the angle of incidence over a period of 50 time steps (1s in real time), before and after the middle point *t*_*m*,*i*_, with a window of 5 time steps (0.1s in real time), respectively. Then we set the threshold values as the maximum values of the absolute moving average. Doubling the length of the period of time over which the average is computed, or doubling the width of the window, do not affect the results. Finally, the time at which the collective U-turn starts (resp. ends) is defined by $\min{\left\{t_{s,i}\right\}}_{i=1}^{N}$ (resp. $\max{\left\{t_{e,i}\right\}}_{i=1}^{N}$).

### Position rank in a group

The relative position of a fish *F*_*i*_ in a group of *N* fish is calculated by projecting the vector position of the fish ${\vec{u}}_{i}$ on the average group velocity vector $\vec{z}=\left(1/N\right)\sum_{i=1}^{N}{\vec{v}}_{i}$. This allows us to define a group centroid in the direction of $\vec{z}$, with respect to which the fish are ranked: the first fish in the group is the fish whose projection on $\vec{z}$ is the most advanced one in the direction of motion of the group (given by $\vec{z}$), the second fish in the group is the second most advanced, and so on. Relative distance between fish are not taken into account when establishing the rank.

### Optimal setting parameters for influential neighbors identification

Four parameters are used to identify influential neighbors: the time-delay *τ*, the window size *w*, the correlation threshold *C*_min_ above which individuals are supposed to be interacting, and the threshold *ε* for selecting more than one influential fish.

The time delay must be specified along the whole trajectory of the focal fish: it is thus a series of values ${\left\{\tau_{k}^{*}\right\}}_{k=0}^{M}$, where *M* is the number of time-steps or frames in the individual U-turn. The parameters *C*_min_, *ε* and *w* are in turn given for all time and for all fish by means of a sensitivity analysis described in the next section.

Assume by now that the three values *C*_min_, *ε* and *w* are known, and denote by *F*_*i*_ the focal fish and by *F*_*j*_ one of its neighbors. Then, the series of time-delays ${\left\{\tau_{k}^{*}\right\}}_{k=0}^{M_{i}}$ is built recursively as follows (actually only *w* is required to extract the time delays).

Denote by *Γ*_*i*_(*t*_*k*_) the highest value of the pairwise directional correlation *C*_*ij*_ of the velocity of fish *F*_*i*_ at time *t*_*k*_ with the velocity of *F*_*j*_ at each time-step in the range of the previous $\left(\tau_{k-1}^{*}+1\right)$ time-steps $R_{k}=\left[0,\tau_{k-1}^{*}+1\right]$:

$$\begin{array}{c} \Gamma_{i}\left(t_{k},w\right)=\max_{\tau_{r}\in R_{k}}\{C_{ij}\left(t_{k},\tau_{r},w\right)\}. \end{array}$$ (6)

Then, the time-delays $\tau_{k}^{*}$, *k* = 1, …, *M*_*i*_, are determined by the smallest value of the time-delay *τ*_*r*_ ∈ *R*_*k*_ where *Γ*_*i*_(*t*_*k*_, *w*) reaches its maximum. For *t*_1_, the maximum correlation is reached at $C_{ij}\left(t_{1},\tau_{1}^{*},w\right)$, for some time-delay $\tau_{1}^{*}\in R_{1}=\left[0,\tau_{0}^{*}+1\right]$. We set $\tau_{0}^{*}=50$ for the initial value of the recurrence. For the rest of time-delays $\tau_{k}^{*}$, *k* = 2, …, *M*_*i*_, the size of *R*_*k*_ is based on the assumption that if, at some time *t*, *F*_*i*_ copies the behavior that *F*_*j*_ displayed at a previous time *t* − *τ*, then, after time *t*, *F*_*i*_ will not copy the behavior that *F*_*j*_ displayed at any time earlier than *t* − *τ*.

Time-delays obtained with more complicated and time consuming procedures such as the time-ordered technique developed in [26] or through the similarity analysis based on Fréchet distances [25] would in principle produce similar values.

**Fig 13:**
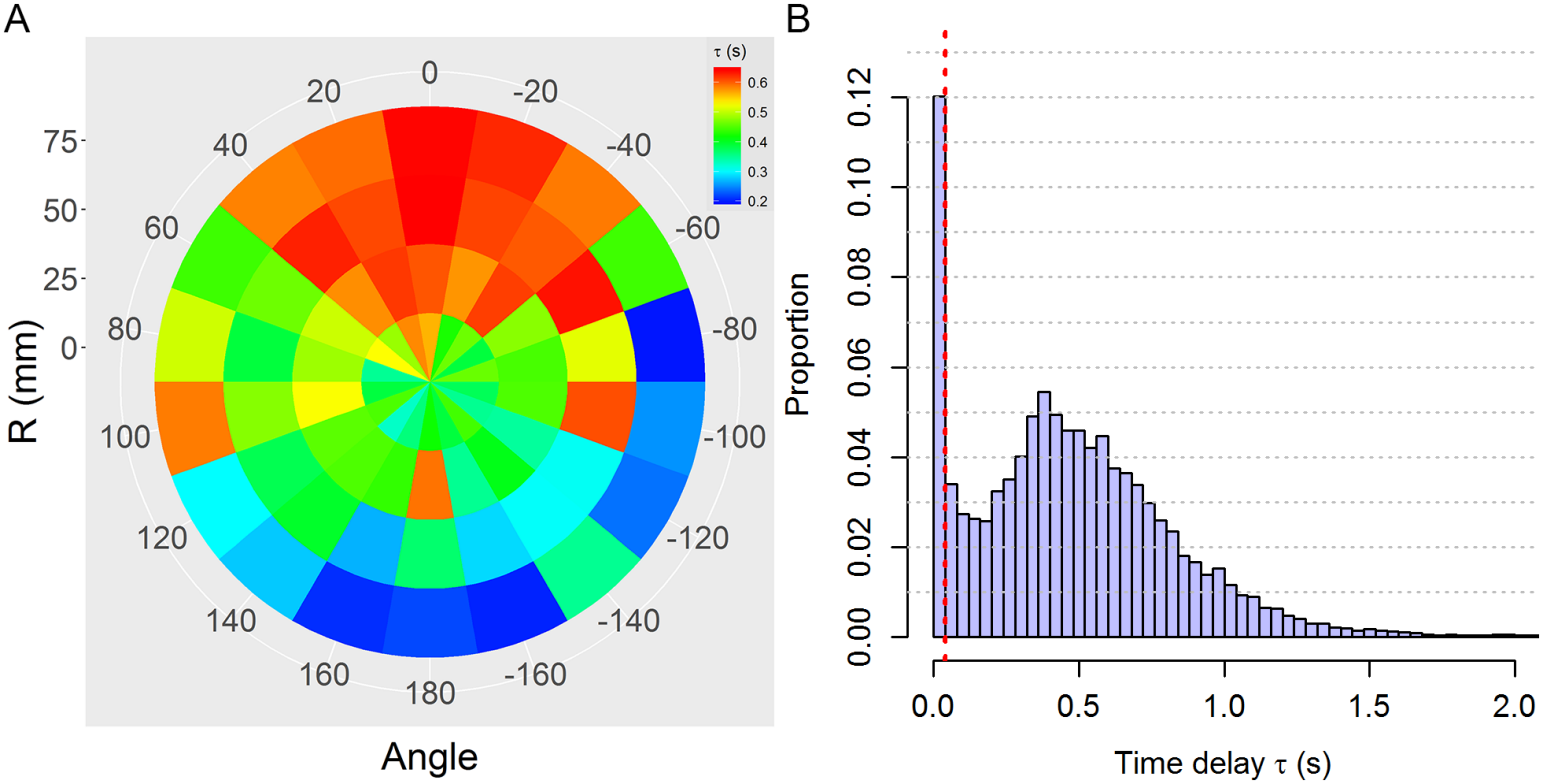
Distribution of time-delay *τ*. (A) Spatial distribution of time-delays obtained by selecting the maximum of the pairwise correlation between the focal fish and its neighbor. The color of each bin represent the mean value of all the cases in that bin. An angle of 0° degree corresponds to when the influential neighbor is in front of the focal individual. (B) Spatially integrated distribution of time-delays. The data here are the same as those used in panel A. The dotted line corresponds to the reaction time threshold *τ*_R_ = 0.04s.

Fig 13B shows the distribution of time-delays obtained with this procedure in groups of two fish. The distribution is clearly bimodal with a first peak when *τ* = 0 and a second one around *τ* = 0.4 s. Considering a reaction time threshold of 50-100 ms for a fish to integrate information and reach a decision [42], we cannot attribute small values of time-delays to situations where the behavioral decision of the focal fish has been influenced by its neighbors. This is confirmed by the analysis of the spatial distribution of the extracted time-delays (Fig 13A), where we show that the lowest average values of *τ* are found mostly when the neighbor was behind the focal fish, in a zone with the lowest perception [15], while the highest values of *τ* > 0.4 s are found when the neighbor is located in front of the focal fish. This has lead us to consider in our analyzes only situations where *τ* > *τ*_R_ = 0.04 s.

### Parameter selection

Although the time-delays ${\left\{\tau_{k}^{*}\right\}}_{k=0}^{M}$ are determined once *w* is known, they also strongly depend on *C*_min_ and *ε*, as the value of these three parameters must be fixed at the same time. This is done by means of a sensitivity analysis in which we have tested the following 40 combinations of parameter values: *w* ∈ {0, 1, 2, 3, 4}, *ε* = {3, 5}, and *C*_min_ ∈ {0.995, 0.99, 0.95, 0.5}.

Each combination (*C*_min_, *ε*, *w*) gives rise to four histograms like those depicted in Fig 7. These histograms constitute the solution of our method of analysis, and can be characterized by a vector $\vec{S}\left(C_{\text{min}},\varepsilon ,w\right)$ in 19 dimensions: (*i*) the 5 proportions of the number of influential neighbors in groups of 5 fish, (*ii*) the 4 proportions of their distance rank, (*iii*) the 5 proportions of their position rank, and (*iv*) the 5 proportions of their turning rank. This allows us to determine how similar are the results arising from two combinations (*C*_min_, *ε*, *w*) and $\left(C_{\text{min}}^{\prime },\varepsilon^{\prime },w^{\prime }\right)$, by computing the cosine similarity of the two vectors $\vec{S}\left(C_{\text{min}},\varepsilon ,w\right)$ and $\vec{S^{\prime }}\left(C_{\text{min}}^{\prime },\varepsilon^{\prime },w^{\prime }\right)$.

The cosine similarity of two vectors $\vec{a}$ and $\vec{b}$, denoted $\cos_{\text{sim}}\left(\vec{a},\vec{b}\right)$, is the cosine of the angle between these two vectors. Thus, two colinear vectors are such that $\cos_{\text{sim}}\left(\vec{a},\vec{b}\right)=\pm 1$ independently of their magnitude, while two perpendicular vectors are such that $\cos_{\text{sim}}\left(\vec{a},\vec{b}\right)=0$. In our case, the components of the vectors are positive, so $\cos_{\text{sim}}\left(\vec{S},\vec{S^{\prime }}\right)\geq 0$ for all (*C*_min_, *ε*, *w*) and $\left(C_{\text{min}}^{\prime },\varepsilon^{\prime },w^{\prime }\right)$. Moreover, as the components are proportions, colinearity implies identity, both in direction and magnitude. Thus, $\cos_{\text{sim}}\left(\vec{S},\vec{S^{\prime }}\right)=1$ means that both results are identical, while $\cos_{\text{sim}}\left(\vec{S},\vec{S^{\prime }}\right)=0$ means that they differ as much as possible.

S5 Fig shows the cosine similarity matrix for the 40 combinations we have tested. Note that the matrix is symmetric with respect to the diagonal, where $\cos_{\text{sim}}\left(\vec{S},\vec{S}\right)=1$. Except for *C*_min_ = 0.5, all similarity values are in the thin range [0.96, 1], showing that all combinations yield practically the same results. The higher dissimilarity is found in the white-yellow lines, where one of the combinations is (*C*_min_, *ε*, *w*) = (0.5, 3, 2).

The selection of parameter values is thus done as follows.

We choose *w* = 2, which corresponds to the higher dissimilarity regions. The selected time window size is sufficiently large so that the jagged nature of the movement data is smoothed out but not too large so that the actual turns gets washed out from the data.

Using *ε* = 3 or *ε* = 5 yields very similar results and we have arbitrarily chosen *ε* = 3.

The selection of *C*_min_ is done by a specific procedure, which consists in calculating the number of data points that remain available for our analysis for each value of *C*_min_. S6 and S7 Figs exhaustively demonstrate that the larger *C*_min_ is, the less data points remain available, and *vice versa*. We might be prone to choose a sufficiently small *C*_min_ in order to get the maximum number of data points. However, according to our definition of influential neighbor, *C*_min_ should be sufficiently large to select only the real influential neighbors. We have thus chosen the highest value which provides a sufficiently large number of data points, that is, the largest value before the fall of the number of data points in S11 Fig, *C*_min_ = 0.95. This value preserves 61% (23830) and 76% (69703) of data points for *N* = 2 and *N* = 5 respectively.

### Null model of collective U-turns

We want to design artificial collective U-turns in groups of 5 fish where all fish perform an individual U-turn at more or less the same place and more or less the same time, and in the same direction (clockwise or anti-clockwise). Fish must coincide in time and space to constitute a “group”, but individual U-turns must happen in an absolutely independent way. Correlations at hand in this paper are thus reduced to a minimum, while preserving the general aspect of a group of fish changing direction.

Our experimental data provide us with 5 × 475 = 2375 trajectories of individual fish, which we have conveniently normalized and combined to build 1000 groups of 5 fish changing direction in the same spatiotemporal interval. This is done as follows.

The whole trajectory of a fish *F*_*i*_ during a U-turn takes place in an interval of time [*t*_*s*,*i*_, *t*_*e*,*i*_], where *t*_*s*,*i*_ is the instant at which the individual U-turn of fish *F*_*i*_ starts, and *t*_*e*,*i*_ is the time at which the individual U-turn ends. See the paragraph above Eq (1). The trajectory of fish *F*_*i*_ in radial coordinates is given by

$$\begin{array}{c} {\left\{\left(\rho_{i}\left(t_{k}\right),\psi_{i}\left(t_{k}\right)\right)\right\}}_{k=1}^{N_{i}}, \end{array}$$ (7)

where *ρ*_*i*_(*t*_*k*_) is the radius (distance of the fish from the center of the tank), *ψ*_*i*_(*t*_*k*_) the already defined angle position (computed anticlockwise as positive), and *N*_*i*_ is the number of time-steps *t*_*k*_ in the trajectory.

Denote by *T*_*i*_ the instant at which fish *F*_*i*_ effectively turns, *i.e.*, *F*_*i*_ is perpendicular to the wall: sin(*θ*_*wi*_(*T*_*i*_)) = 0. In well defined individual U-turns as the ones we are using in our data, this happens only once per U-turn. Accordingly, (*ρ*_*i*_(*T*_*i*_), *ψ*_*i*_(*T*_*i*_)) denotes the fish position at time *T*_*i*_.

Although we would like to have absolutely uncorrelated fish, it would not make sense to use groups of trajectories that do not reproduce a consistent U-turn, *e.g.*, if one fish makes its U-turn much later than another, or on the other side of the tank. We thus try to decorrelate fish trajectories as much as possible, while preserving at the same time the typical spatiotemporal shape of real collective U-turns.

The decorrelation of all individual U-turns is done with the following two steps:

- Spatial rotation: For all individual fish *F*_*i*_ in all U-turns, we rotate its trajectory an angle −*ψ*_*i*_(*T*_*i*_) + *π*/2 + *ξ*_*i*_, where *ξ*_*i*_ is a random number in [−*π*/12, *π*/12] sampled uniformly, so that the new location of fish *F*_*i*_ at the time *T*_*i*_ when it performs its individual U-turn is in the upper part of the tank around *π*/2, in [5*π*/12, 7*π*/12].
- Time shift: For all individual fish *F*_*i*_ in all U-turns, we shift the time scale a value −*T*_*i*_ + *ζ*_*i*_, where *ζ*_*i*_ is a random number sampled uniformly in [−1, 1] s, so that *F*_*i*_ makes its individual U-turn at around time 0, in [−1, 1] seconds.

The artificial collective U-turn is thus built as follows:

1. Select randomly 5 real collective U-turns, and, from each collective U-turn, select randomly one trajectory. Rotate and time-shift trajectories according to the process described above.
2. Select randomly one of the 5 fish as the fish of reference *F*_ref_ for building the artificial U-turn. If necessary, mirror the trajectories of other fish so that all fish move in the same direction as *F*_ref_ with respect to the center of the tank, *i.e.*, clockwise or anti-clockwise.

Then, the fish of reference of the artificial U-turn will make its individual U-turn at time *ζ*_ref_ ∈ [−1, 1] s and position (*ρ*_ref_(*T*_ref_), *π*/2 + *ξ*_ref_). The other four fish *F*_*j*_ will make their individual U-turn at time *ζ*_*j*_ ∈ [−1, 1] s and position (*ρ*_*j*_(*T*_*j*_), *π*/2 + *ξ*_*j*_) respectively, for *j* = 1, …, 5, *j* ≠ ref.

We have depicted in S9 Fig a set of artificial U-turns for comparison with the real U-turns shown in S8 Fig. Note that in these figures the time-scale has been shifted again so that collective U-turns start at *t* = 0 s.

## Supporting information

S1 Video Sample video of an U-turn event in a group of 5 fish. Original video of an U-turn event, corresponding to Fig 4 and S2 Video.
(AVI)

S2 Video Sample video of an U-turn dynamic in a group of 5 fish. Video showing the velocities of fish and interaction dynamics in the group, corresponding to Fig 4 and S1 Video.
(AVI)

S1 Fig Directional correlation *H*_*ij*_(*t*, *τ*) between fish *F*_*i*_ and *F*_*j*_. For *i* = 2, …, 5 (rows) and *j* = 1, …, 5, *j* ≠ *i* (columns), *e.g.*, first row is for fish *F*_2_: (A) *H*_21_(*t*, *τ*), (B) *H*_23_(*t*, *τ*), (C) *H*_24_(*t*, *τ*) and (D) *H*_25_(*t*, *τ*).
(TIF)

S2 Fig Different values of τ* for different subsets of the same data set computed with the method of Nagy *et al*. [23]. Consider the dataset of U-turns of 2 fish composed by U-turn number 1 to U-turn number 36, coming from the same experiment, and divide it in two subsets *S*_*A*_ and *S*_*B*_ containing respectively the U-turns [1,…,18] and the U-turns [19,…,36]. (A) Average directional correlation *C*_*ij*_ with respect to time-delay *τ* for the U-turns from dataset *S*_*A*_. Red star and dashed blue vertical line denotes τ* = 0.96. (B) *C*_*ij*_ for the U-turns from dataset *S*_*B*_. Red star: τ* = 0.32. (C) *C*_*ij*_ for all the U-turn in data set *S*_*A*_ ∪ *S*_*B*_. Red star: τ* = 0.80. The method of Nagy *et al*. is based on the assumption that the pairwise interaction between two individuals in a group has a constant time-delay τ*. However, Panels A and B provide different values of τ* for different data sets, showing that the method of Nagy *et al*. is not suitable for studing our data, and that the method we introduce here, which is based on the detection of dynamic time-delays, has potential for a broader range of applications.
(TIF)

S3 Fig Distribution of the average duration (in seconds) of (A) individual and (B) collective U-turns in groups of 2 fish. Collective U-turns last around twice the duration of individual U-turns.
(TIF)

S4 Fig Distribution of the average duration (in seconds) of (A) individual and (B) collective U-turns in groups of 5 fish. Collective U-turns last almost four times the duration of individual U-turns.
(TIF)

S5 Fig Parameter comparison matrix. Matrix of 40 × 40 square cells, where each cell corresponds to the similarity value SV arising from the comparison of the two parameter combinations shown in the corresponding horizontal and vertical axes. We considered 40 parameter combinations, thus the size of the matrix. The similarity value SV is represented by the color of the cell, where the brightest red color corresponds to SV = 1 and the white color to SV = 0.92. For instance, the top-left cell displays a similarity value of SV = 0.95, showing how similar the results are when comparing the two combinations {*ε* = 5, *C*_min_ = 0.995, *w* = 0} (horizontal axis) and {*ε* = 3, *C*_min_ = 0.5, *w* = 4} (vertical axis). Cells along the diagonal correspond to the comparison of two identical parameter combinations and therefore SV = 1 there.
(TIF)

S6 Fig Available data for different values of the average directional correlation threshold *C*_min_ in the case of *N* = 2 fish. **Small panels:** (there are 10, one per experiment) Number of data points available from the respective experiment for each value of *C*_min_ in [0.5, 1]. The values of *C*_min_ are denoted by small circles. Three specific values are shown by arrows: 0.6, 0.95 and 0.995. The value highlighted in red corresponds to the value we chose and is denoted by a star instead of a circle. Each vertical line corresponds to the fish that is taken as being the focal fish: *F*_1_ (red) and *F*_2_ (cyan). For instance, selecting *C*_min_ = 0.6 in the upper-left small panel, 700 data points will be available for both fish. For *C*_min_ = 0.95, around 450 points will be available for both fish.
**Leftmost higher panel:** Total number of data points available from all fish from all the experiments (summary of the 10 small panels, *i.e.*, there is only one –pink– line). Vertical axis: ratio between the available number of data points for *C*_min_ and the number of data points available for *C*_min_ = 0.5. Total data points available from all the experiments (for *C*_min_ = 0.5): 39381; data points available for *C*_min_ = 0.95: 23830.
(TIF)

S7 Fig Available data for different values of the average directional correlation threshold *C*_min_, in the case of *N* = 5 fish. **Small panels:** (there are 10, one per experiment) Number of data points available from the respective experiment for each value of *C*_min_ in [0.5, 1]. The values of *C*_min_ are denoted by small circles. Three specific values are shown by arrows: 0.6, 0.95 and 0.995. The value highlighted in red corresponds to the value we chose and is denoted by a star instead of a circle. Each vertical line corresponds to the fish that is taken as being the focal fish: *F*_1_ (red), *F*_2_ (yellow), *F*_3_ (green), *F*_4_ (blue) and *F*_5_ (magenta). For instance, selecting *C*_min_ = 0.6 in the third small panel of the upper row, 55 data points will be available for each one of the 5 fish. For *C*_min_ = 0.95, around 75 points will be available for each fish.
**Leftmost higher panel:** Total number of data points available from all fish from all the experiments (summary of the 10 small panels, *i.e.*, there is only one –pink– line). Vertical axis: ratio between the available number of data points for *C*_min_ and the number of data points available for *C*_min_ = 0.5. Total data points available from all the experiments (for *C*_min_ = 0.5): 91827; data points available for *C*_min_ = 0.95: 69703.
(TIF)

S8 Fig Collective U-turns observed in experiments with *N* = 5 fish. (TIF)

S9 Fig Artificial collective U-turns obtained with the null model. (TIF)

S10 Fig Homogeneous (isotropic) spatial distribution of “influential neighbors” in collective artificial U-turns. (A) Density map of “influential neighbors” location (blue) and their average relative velocity field (arrows) with respect to the focal fish (red arrow). (B) Average spatial distribution.
(TIF)

S11 Fig Number of available data points for different values of *C*_min_. Solid black line: Remaining data points for each value of *C*_min_ for *N* = 2 according to the leftmost panel in S6 Fig. Red line: same thing, for *N* = 5, according to S7 Fig. Dashed line: highest number of available data points before the sharp fall of the black curve at *C*_min_ = 0.95.
(TIF)
