# Correction: Identifying influential neighbors in animal flocking

**DOI:** 10.1371/journal.pcbi.1005902

**Published:** 2017-12-12

**Authors:** The PLOS Computational Biology Staff

The link to this article’s data is incorrect. The correct link is: https://doi.org/10.6084/m9.figshare.5631988.v1
