# Supplementary figures and images for: Identifying influential neighbors in animal flocking
Source: PLoS Comput Biol. 2017 Nov 21;13(11):e1005822. doi: 10.1371/journal.pcbi.1005822 (PMC5697824; doi:10.1371/journal.pcbi.1005822)

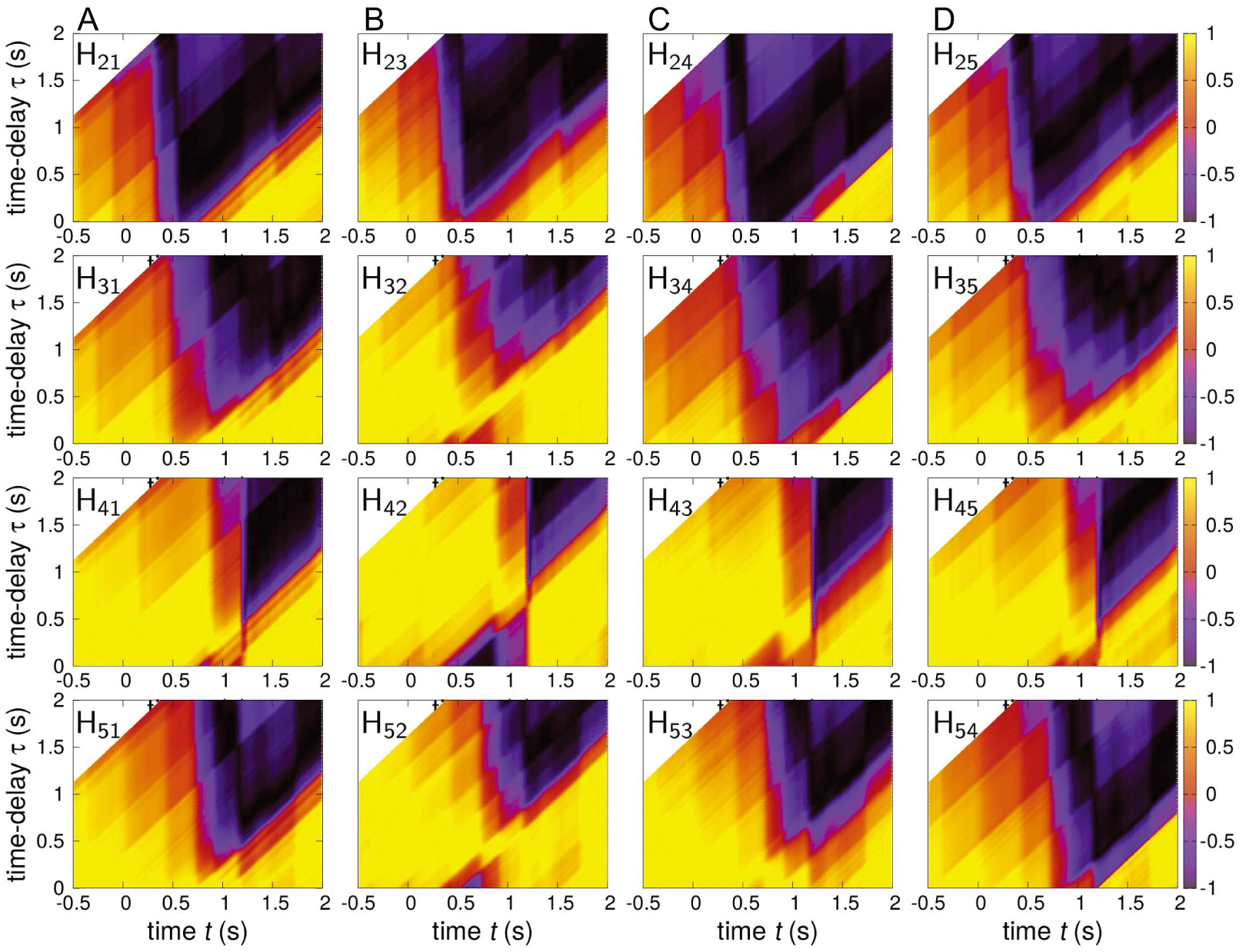

Supplement: S1 Fig — For i = 2, …, 5 (rows) and j = 1, …, 5, j ≠ i (columns), e.g., first row is for fish F2: (A) H21(t, τ), (B) H23(t, τ), (C) H24(t, τ) and (D) H25(t, τ). (TIF) [file pcbi.1005822.s003.tif]

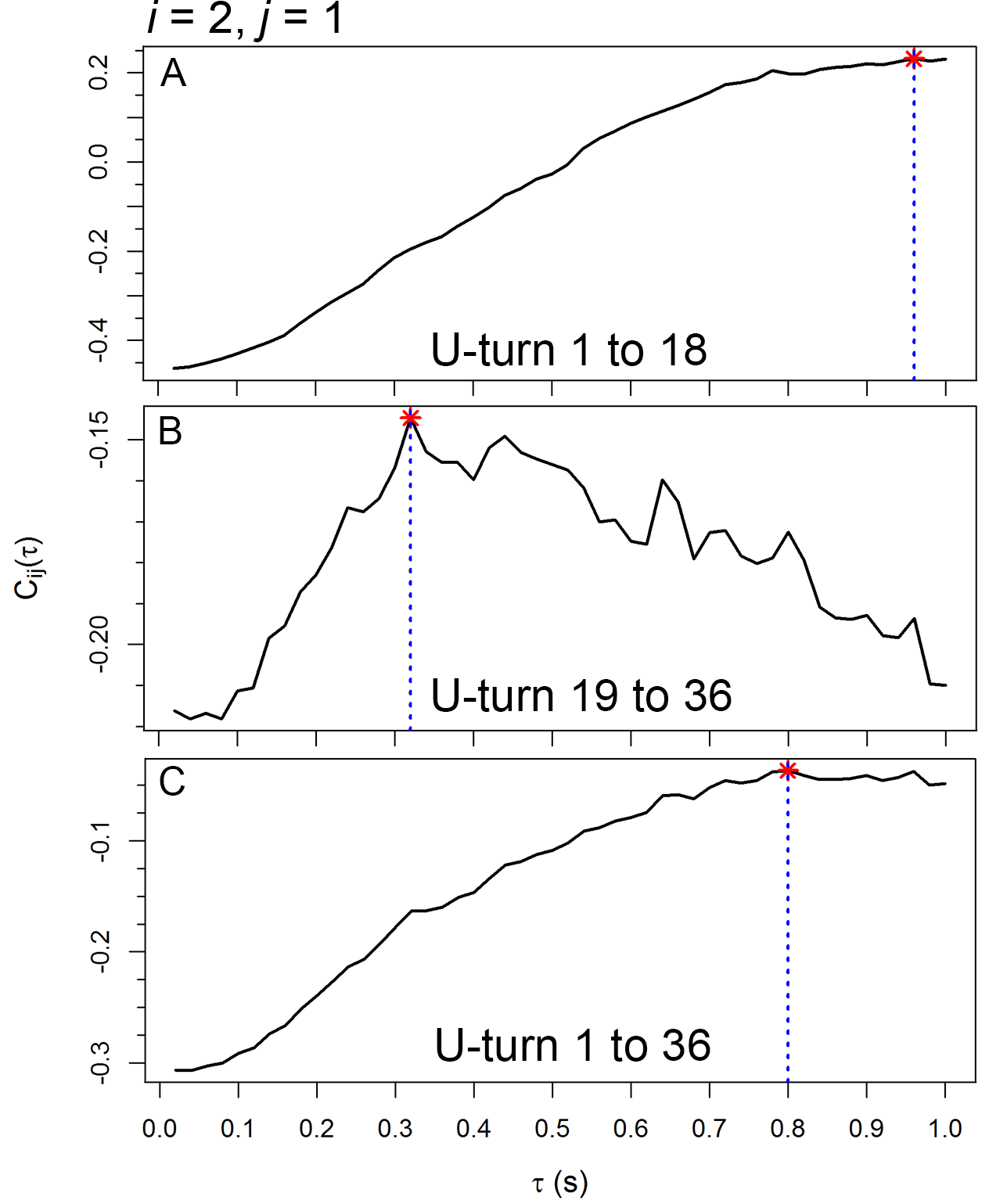

Supplement: S2 Fig — Consider the dataset of U-turns of 2 fish composed by U-turn number 1 to U-turn number 36, coming from the same experiment, and divide it in two subsets SA and SB containing respectively the U-turns [1,…,18] and the U-turns [19,…,36]. (A) Average directional correlation Cij with respect to time-delay τ for the U-turns from dataset SA. Red star and dashed blue vertical line denotes τ* = 0.96. (B) Cij for the U-turns from dataset SB. Red star: τ* = 0.32. (C) Cij for all the U-turn in data set SA ∪ SB. Red star: τ* = 0.80. The method of Nagy et al. is based on the assumption that the pairwise interaction between two individuals in a group has a constant time-delay τ*. However, Panels A and B provide different values of τ* for different data sets, showing that the method of Nagy et al. is not suitable for studing our data, and that the method we introduce here, which is based on the detection of dynamic time-delays, has potential for a broader range of applications. (TIF) [file pcbi.1005822.s004.tif]

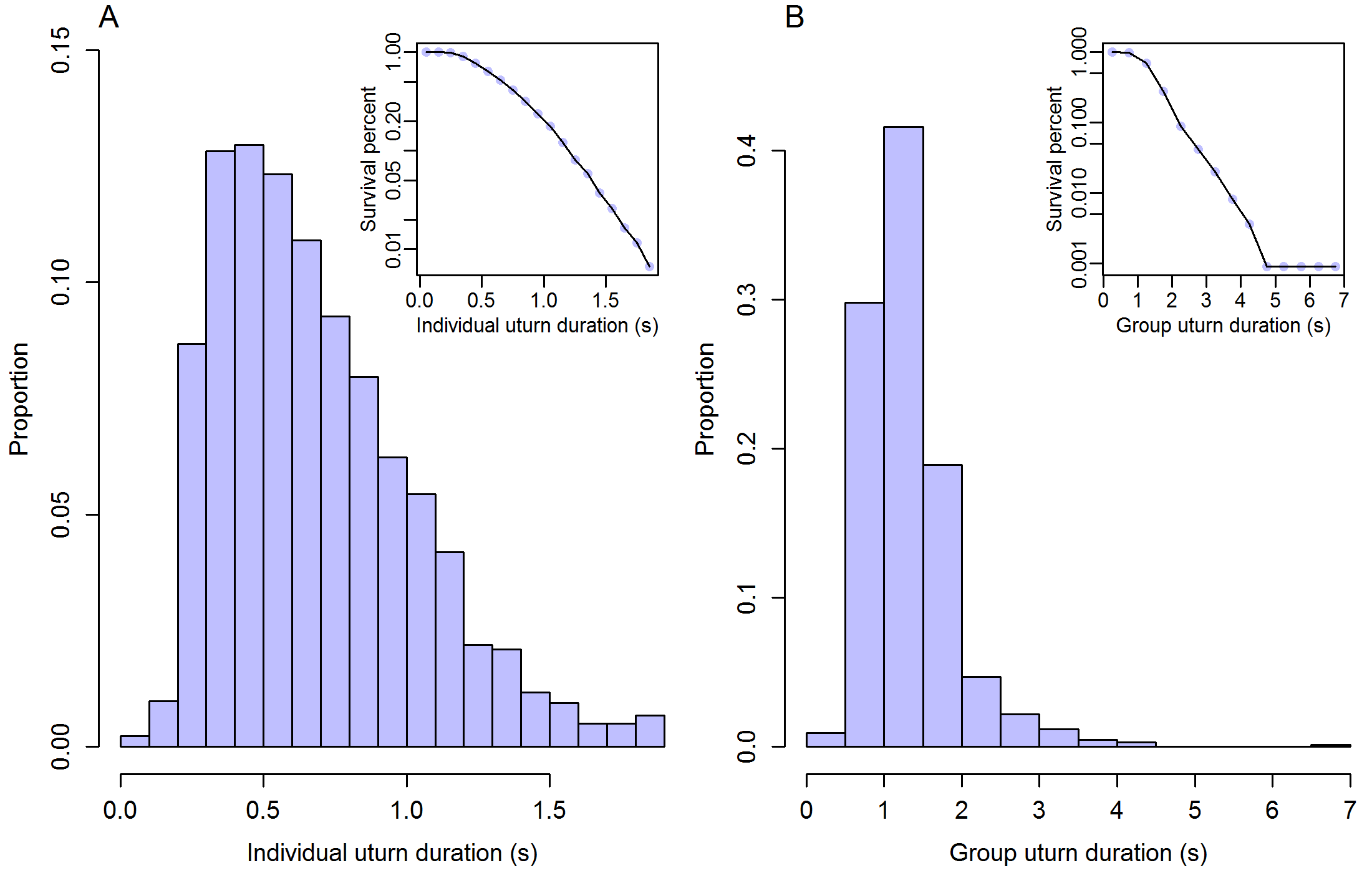

Supplement: S3 Fig — Collective U-turns last around twice the duration of individual U-turns. (TIF) [file pcbi.1005822.s005.tif]

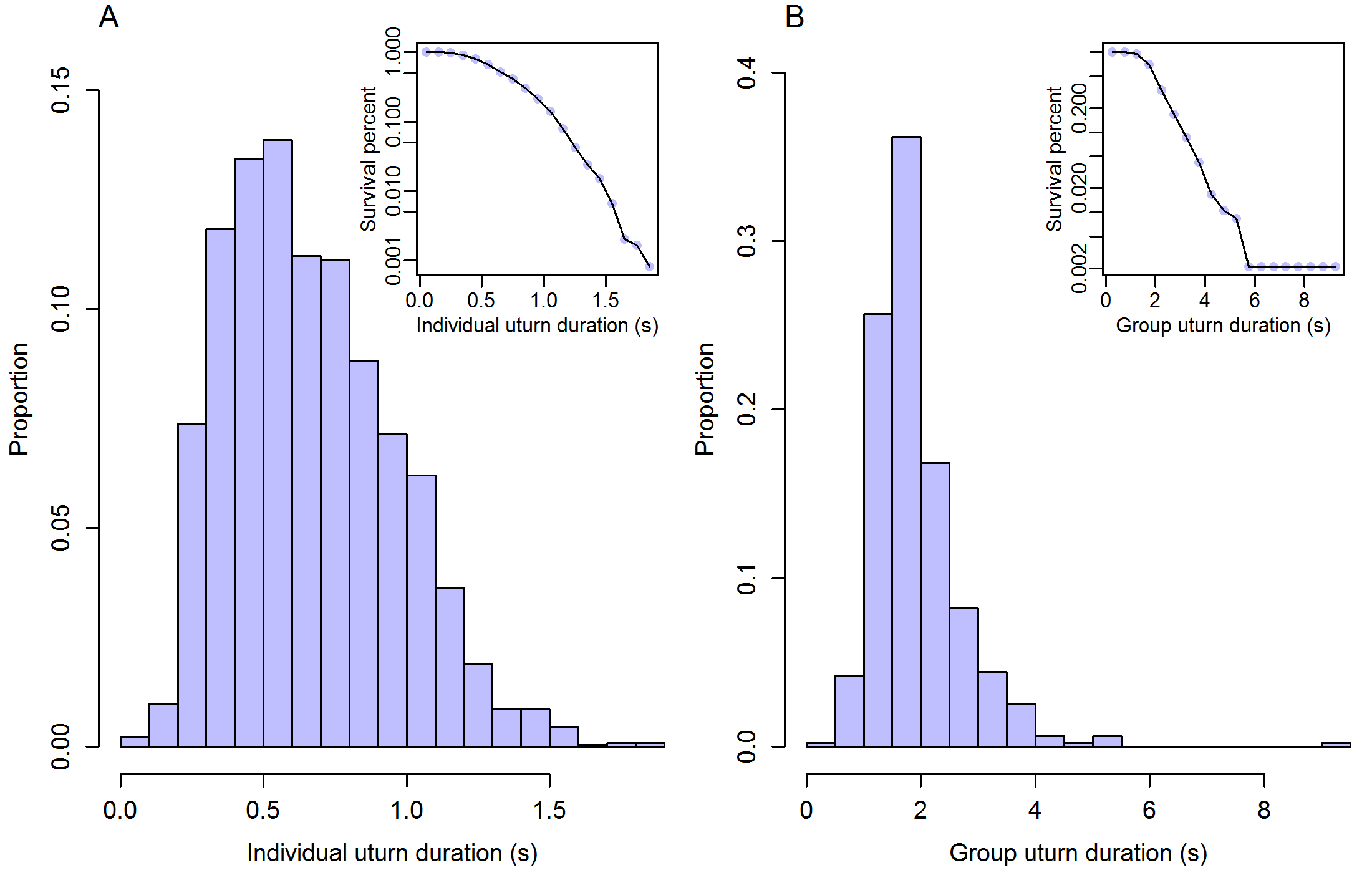

Supplement: S4 Fig — Collective U-turns last almost four times the duration of individual U-turns. (TIF) [file pcbi.1005822.s006.tif]

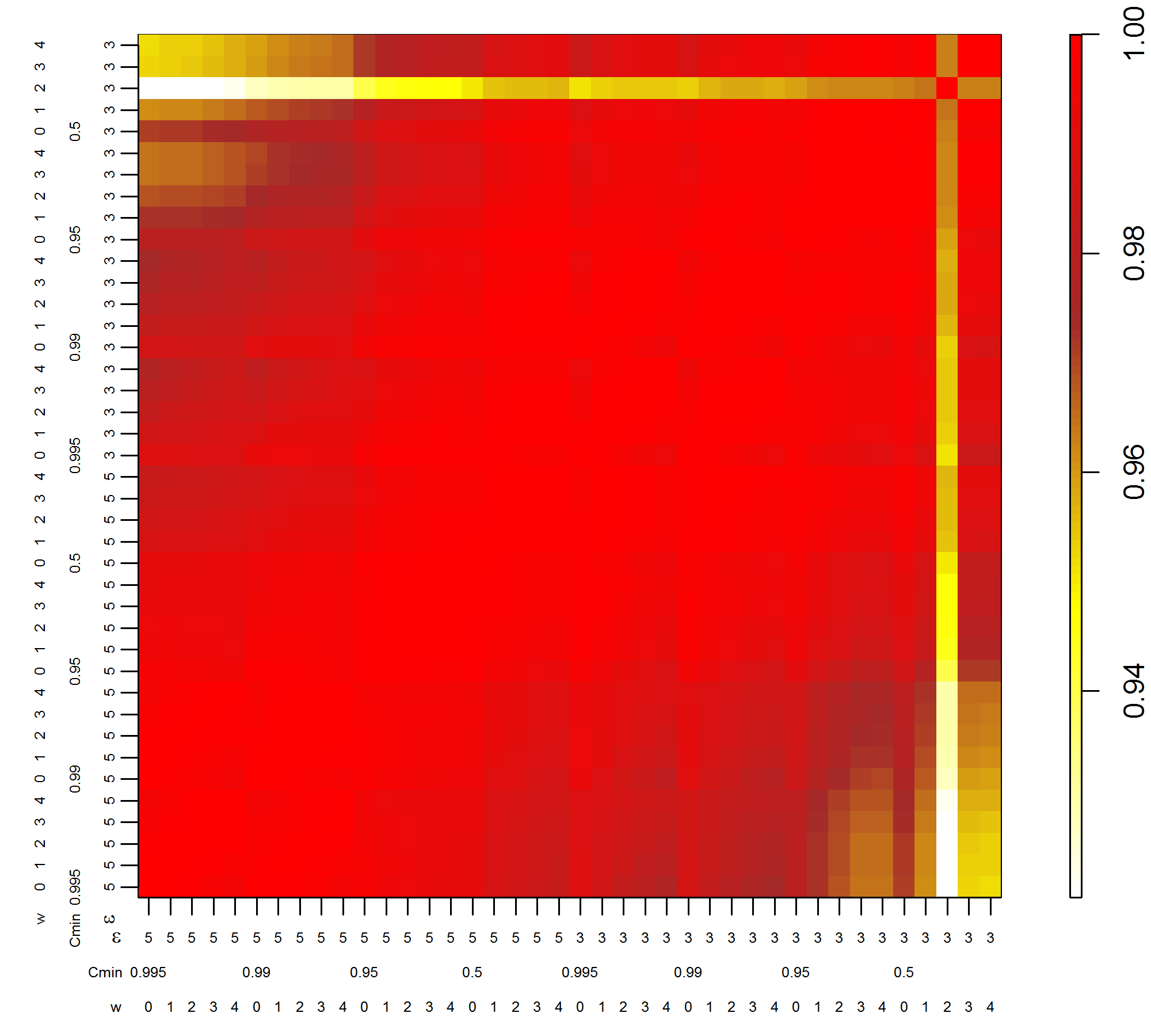

Supplement: S5 Fig — Matrix of 40 × 40 square cells, where each cell corresponds to the similarity value SV arising from the comparison of the two parameter combinations shown in the corresponding horizontal and vertical axes. We considered 40 parameter combinations, thus the size of the matrix. The similarity value SV is represented by the color of the cell, where the brightest red color corresponds to SV = 1 and the white color to SV = 0.92. For instance, the top-left cell displays a similarity value of SV = 0.95, showing how similar the results are when comparing the two combinations {ε = 5, Cmin = 0.995, w = 0} (horizontal axis) and {ε = 3, Cmin = 0.5, w = 4} (vertical axis). Cells along the diagonal correspond to the comparison of two identical parameter combinations and therefore SV = 1 there. (TIF) [file pcbi.1005822.s007.tif]

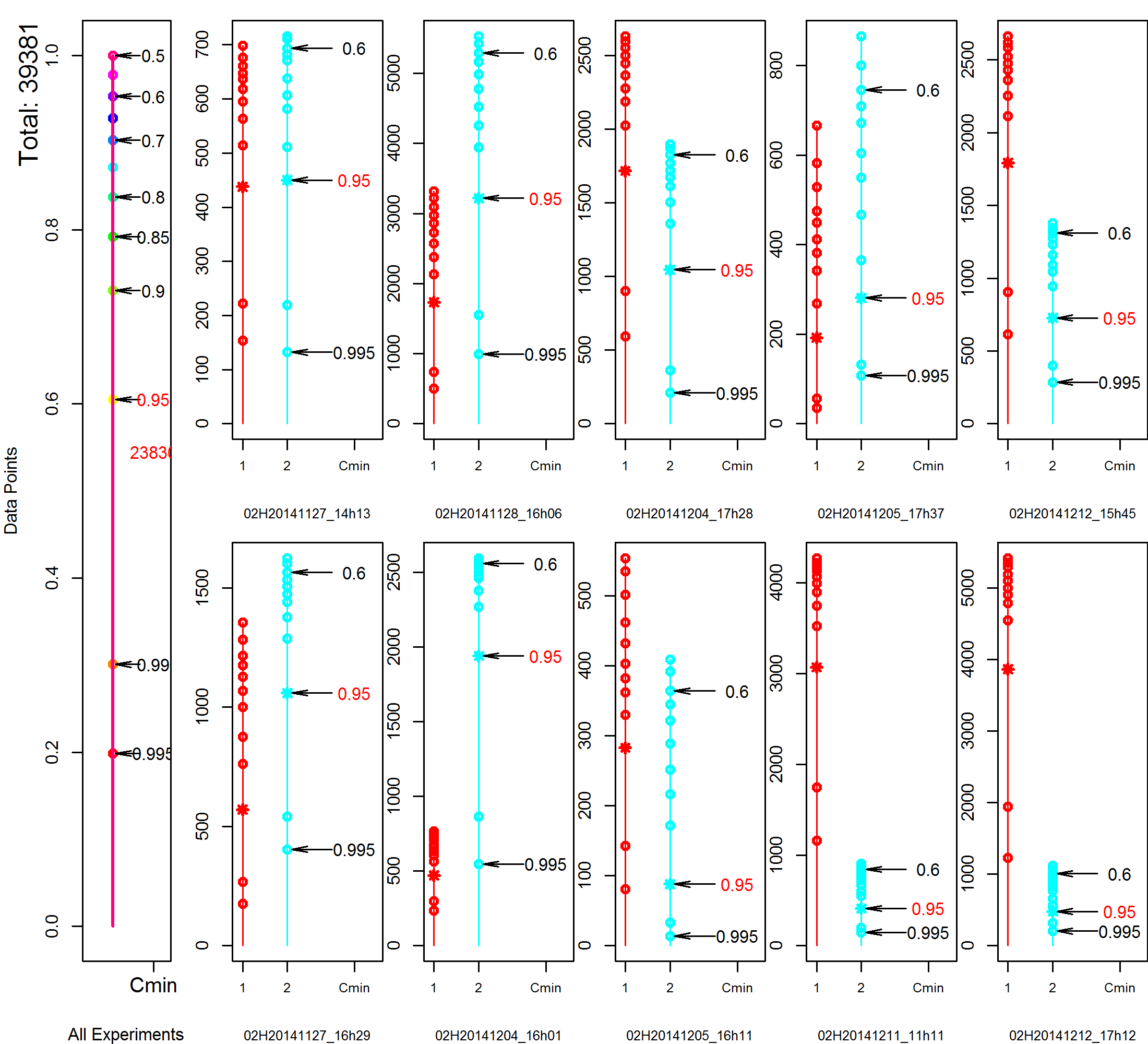

Supplement: S6 Fig — Small panels: (there are 10, one per experiment) Number of data points available from the respective experiment for each value of Cmin in [0.5, 1]. The values of Cmin are denoted by small circles. Three specific values are shown by arrows: 0.6, 0.95 and 0.995. The value highlighted in red corresponds to the value we chose and is denoted by a star instead of a circle. Each vertical line corresponds to the fish that is taken as being the focal fish: F1 (red) and F2 (cyan). For instance, selecting Cmin = 0.6 in the upper-left small panel, 700 data points will be available for both fish. For Cmin = 0.95, around 450 points will be available for both fish. Leftmost higher panel: Total number of data points available from all fish from all the experiments (summary of the 10 small panels, i.e., there is only one –pink– line). Vertical axis: ratio between the available number of data points for Cmin and the number of data points available for Cmin = 0.5. Total data points available from all the experiments (for Cmin = 0.5): 39381; data points available for Cmin = 0.95: 23830. (TIF) [file pcbi.1005822.s008.tif]

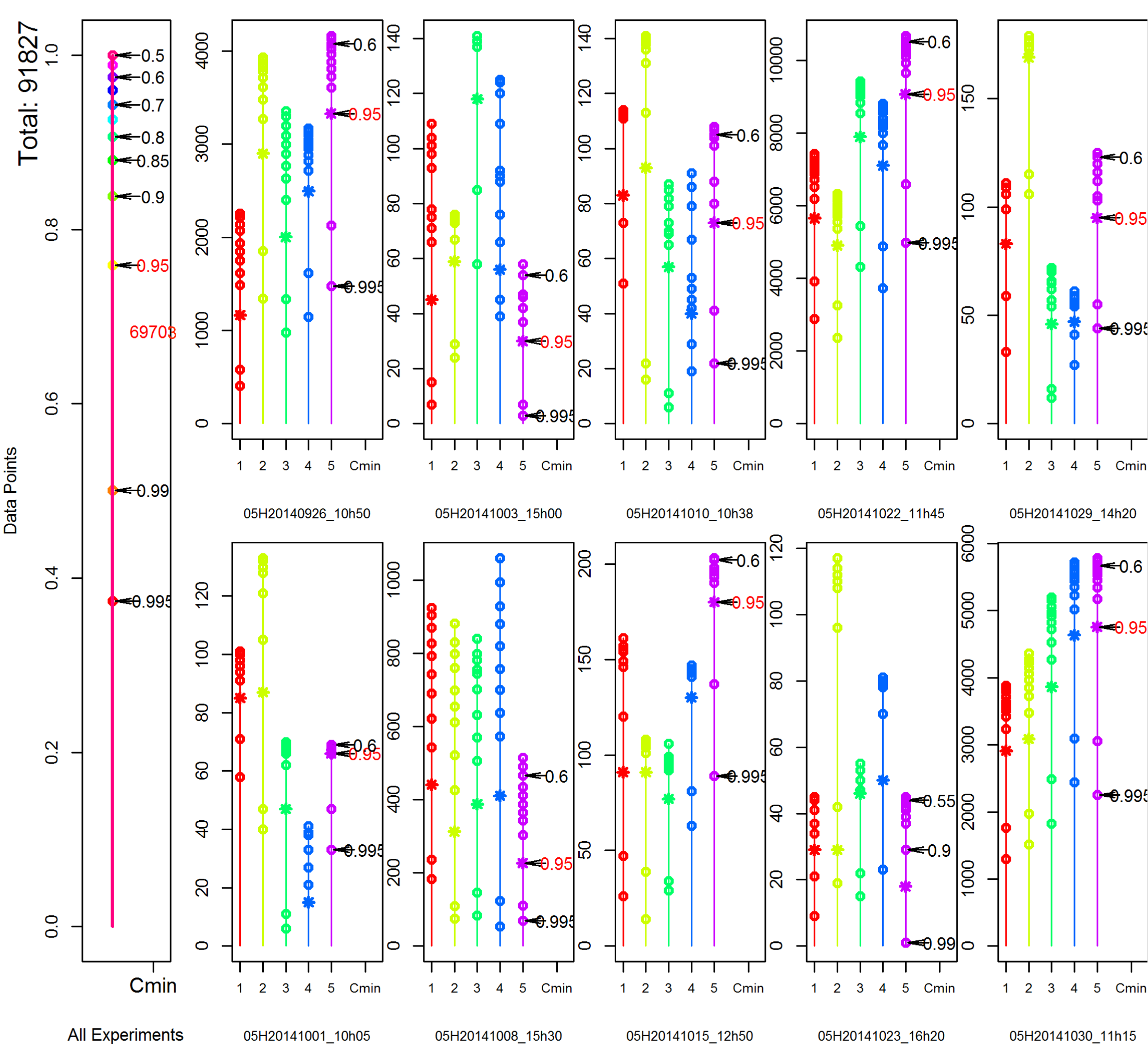

Supplement: S7 Fig — Small panels: (there are 10, one per experiment) Number of data points available from the respective experiment for each value of Cmin in [0.5, 1]. The values of Cmin are denoted by small circles. Three specific values are shown by arrows: 0.6, 0.95 and 0.995. The value highlighted in red corresponds to the value we chose and is denoted by a star instead of a circle. Each vertical line corresponds to the fish that is taken as being the focal fish: F1 (red), F2 (yellow), F3 (green), F4 (blue) and F5 (magenta). For instance, selecting Cmin = 0.6 in the third small panel of the upper row, 55 data points will be available for each one of the 5 fish. For Cmin = 0.95, around 75 points will be available for each fish. Leftmost higher panel: Total number of data points available from all fish from all the experiments (summary of the 10 small panels, i.e., there is only one –pink– line). Vertical axis: ratio between the available number of data points for Cmin and the number of data points available for Cmin = 0.5. Total data points available from all the experiments (for Cmin = 0.5): 91827; data points available for Cmin = 0.95: 69703. (TIF) [file pcbi.1005822.s009.tif]

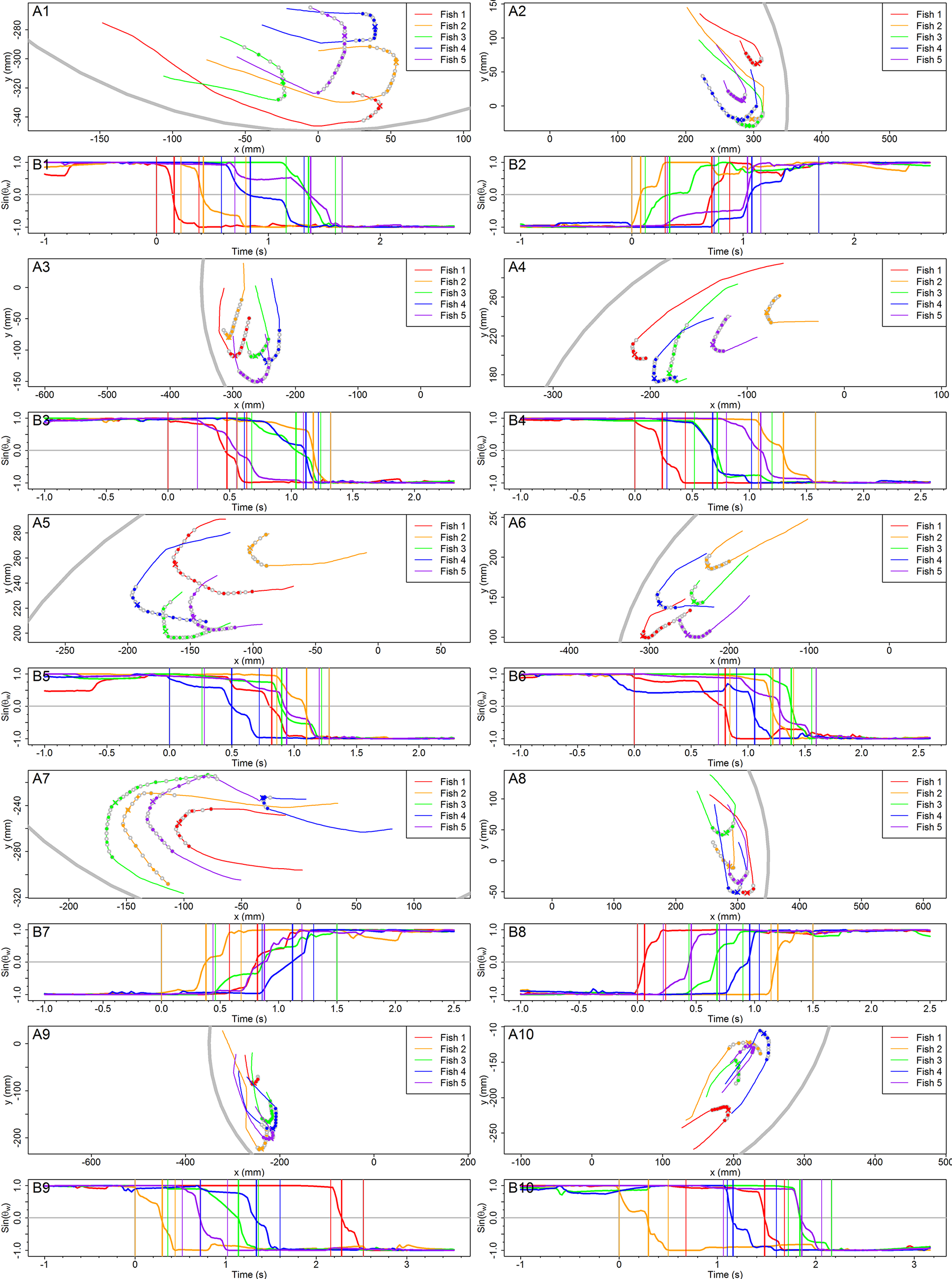

Supplement: S8 Fig — (TIF) [file pcbi.1005822.s010.tif]

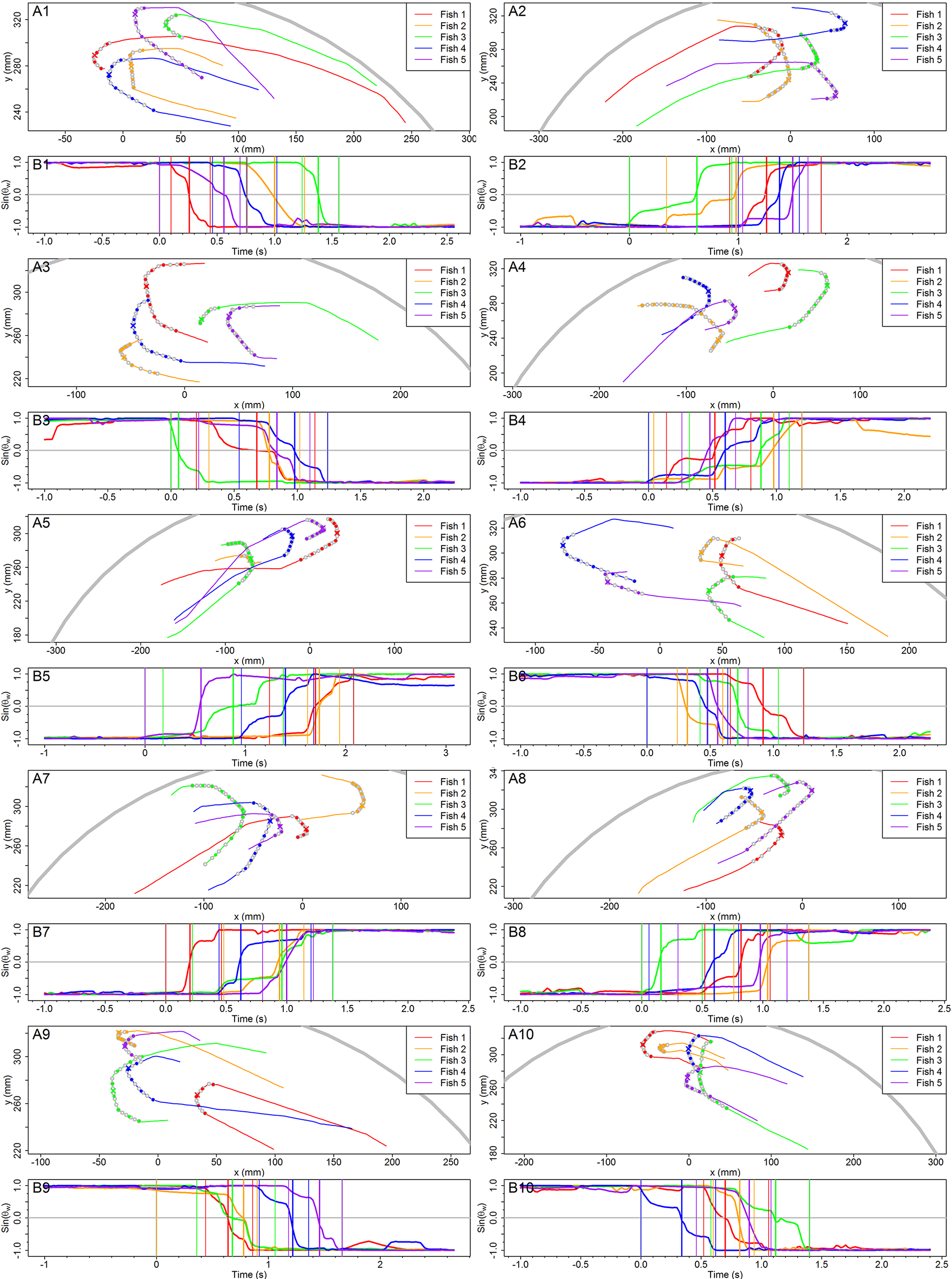

Supplement: S9 Fig — (TIF) [file pcbi.1005822.s011.tif]

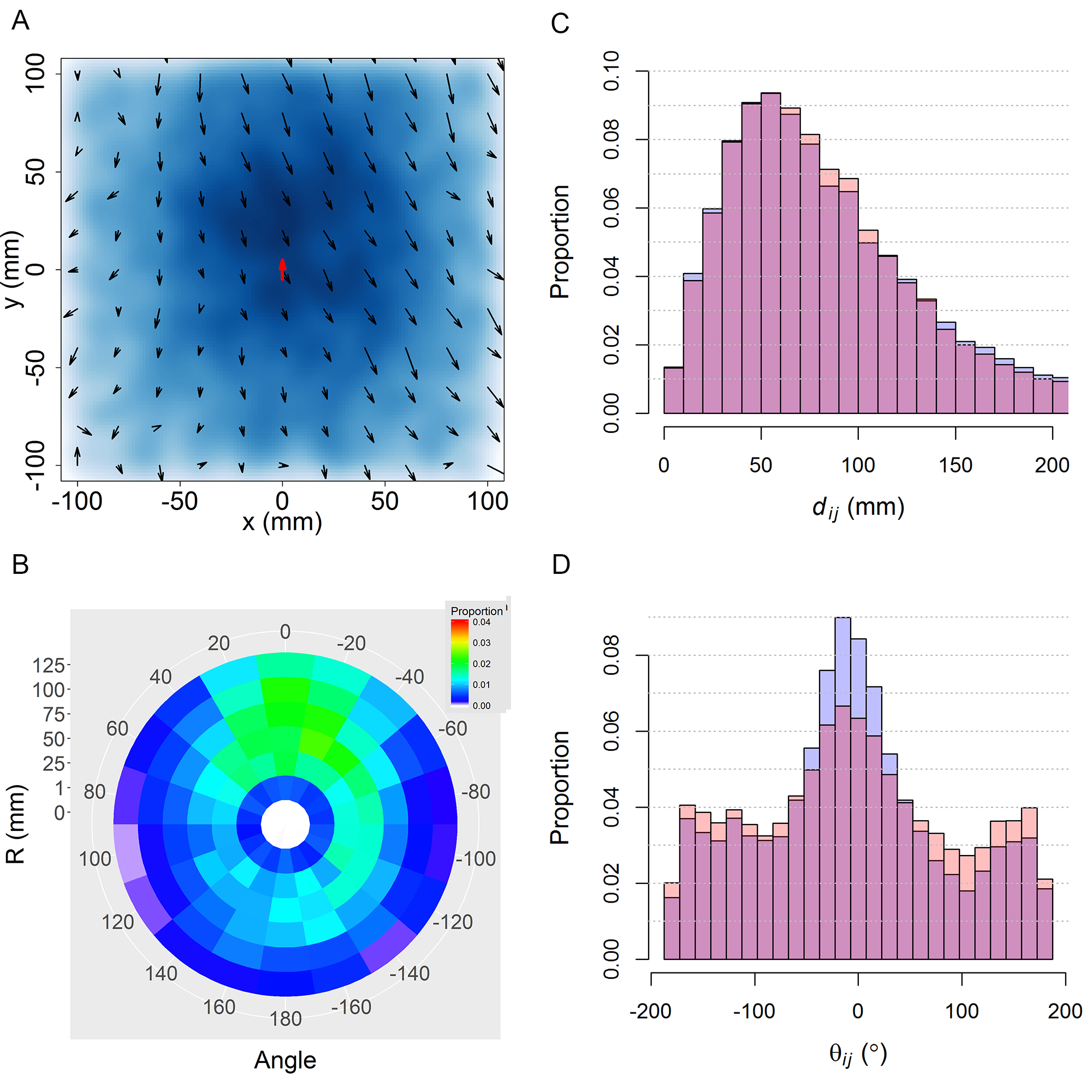

Supplement: S10 Fig — (A) Density map of “influential neighbors” location (blue) and their average relative velocity field (arrows) with respect to the focal fish (red arrow). (B) Average spatial distribution. (TIF) [file pcbi.1005822.s012.tif]

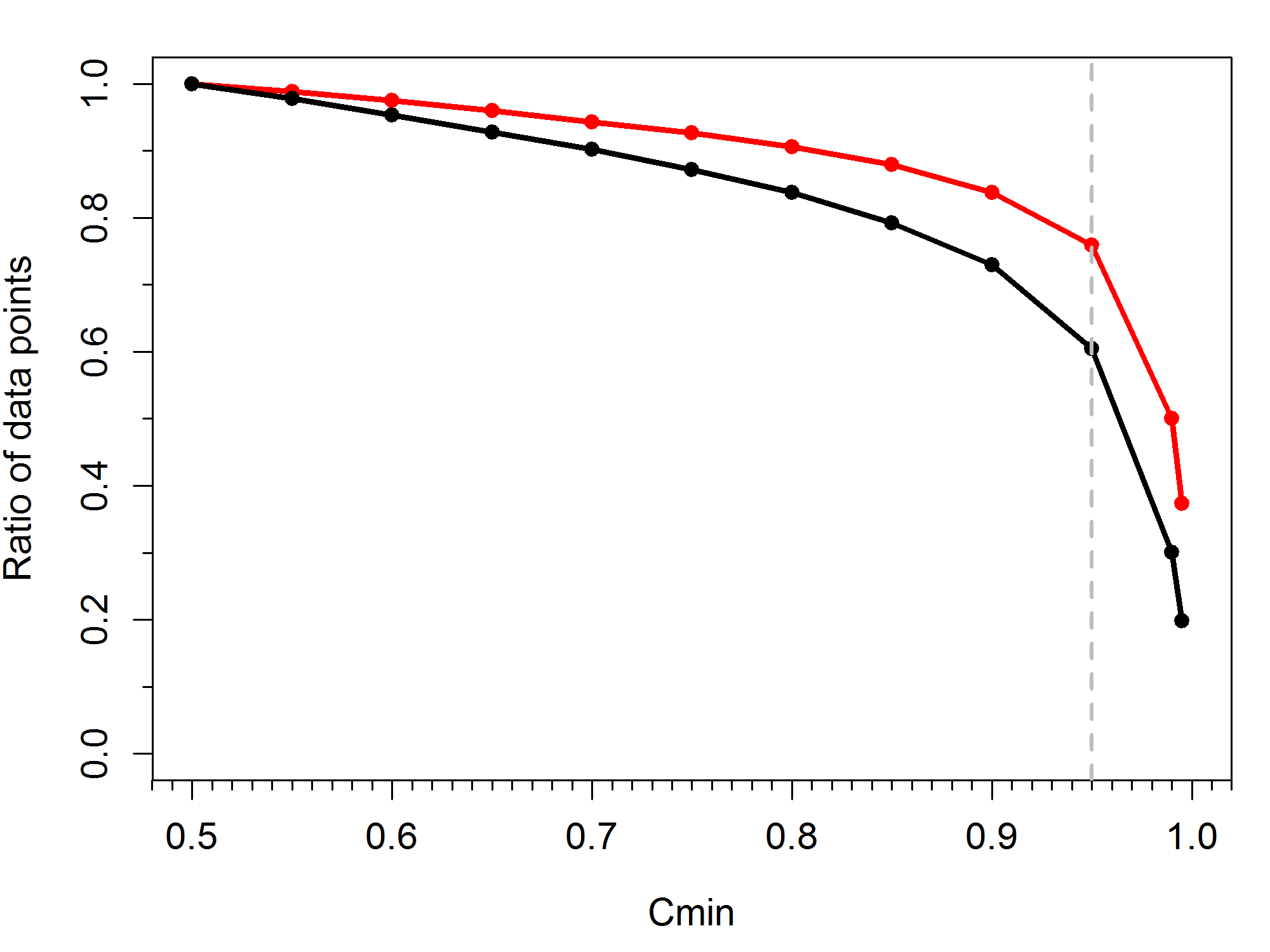

Supplement: S11 Fig — Solid black line: Remaining data points for each value of Cmin for N = 2 according to the leftmost panel in S6 Fig. Red line: same thing, for N = 5, according to S7 Fig. Dashed line: highest number of available data points before the sharp fall of the black curve at Cmin = 0.95. (TIF) [file pcbi.1005822.s013.tif]
